# Functional conservation and divergence of *Miscanthus lutarioriparius* GT43 gene family in xylan biosynthesis

**DOI:** 10.1186/s12870-016-0793-5

**Published:** 2016-04-26

**Authors:** Xiaoyu Wang, Qi Tang, Xun Zhao, Chunlin Jia, Xuanwen Yang, Guo He, Aimin Wu, Yingzhen Kong, Ruibo Hu, Gongke Zhou

**Affiliations:** Qingdao Institute of Bioenergy and Bioprocess Technology, Key Laboratory of Biofuels, Qingdao Engineering Research Center of Biomass Resources and Environment, Chinese Academy of Sciences, Qingdao, 266101 PR China; University of Chinese Academy of Sciences, Beijing, 100049 PR China; Shandong Institute of Agricultural Sustainable Development, Jinan, 250100 PR China; State Key Laboratory for Conservation and Utilization of Subtropical Agrobioresources, South China Agricultural University, Guangzhou, 510642 PR China; Tobacco Research Institute of Chinese Academy of Agricultural Sciences, Key laboratory of Tobacco Genetic Improvement and Biotechnology, Qingdao, 266101 PR China

**Keywords:** *Miscanthus lutarioriparius*, Glycosyltransferase family 43, Xylan biosynthesis, Secondary cell wall, Seed coat mucilage

## Abstract

**Background:**

Xylan is the most abundant un-cellulosic polysaccharides of plant cell walls. Much progress in xylan biosynthesis has been gained in the model plant species *Arabidopsis*. Two homologous pairs *Irregular Xylem 9 (IRX9)/9L* and *IRX14/14L* from glycosyltransferase (GT) family 43 have been proved to play crucial roles in xylan backbone biosynthesis. However, xylan biosynthesis in grass such as *Miscanthus* remains poorly understood.

**Results:**

We characterized seven GT43 members in *M. lutarioriparius*, a promising bioenergy crop. Quantitative real-time RT-PCR (qRT-PCR) analysis revealed that the expression of *MlGT43* genes was ubiquitously detected in the tissues examined. In-situ hybridization demonstrated that *MlGT43A-B* and *MlGT43F-G* were specifically expressed in sclerenchyma, while *MlGT43C-E* were expressed in both sclerenchyma and parenchyma. All seven MlGT43 proteins were localized to Golgi apparatus. Overexpression of *MlGT43A-E* but not *MlGT43F* and *MlGT43G* in *Arabidopsis irx9* fully or partially rescued the mutant defects, including morphological changes, collapsed xylem and increased xylan contents, whereas overexpression of *MlGT43F* and *MlGT43G* but not *MlGT43A-E* complemented the defects of *irx14,* indicating that *MlGT43A-E* are functional orthologues of *IRX9*, while *MlGT43F* and *MlGT43G* are functional orthologues of *IRX14*. However, overexpression of all seven *MlGT43* genes could not rescue the mucilage defects of *irx14* seeds. Furthermore, transient transactivation analyses of *MlGT43A-E* reporters demonstrated that *MlGT43A* and *MlGT43B* but not *MlGT43C-E* were differentially activated by *MlSND1*, *MlMYB46* or *MlVND7*.

**Conclusion:**

The results demonstrated that all seven MlGT43s are functionally conserved in xylan biosynthesis during secondary cell wall formation but diversify in seed coat mucilage xylan biosynthesis. The results obtained provide deeper insight into xylan biosynthesis in grass, which lay the foundation for genetic modification of grass cell wall components and structure to better suit for next-generation biofuel production.

**Electronic supplementary material:**

The online version of this article (doi:10.1186/s12870-016-0793-5) contains supplementary material, which is available to authorized users.

## Highlight

The functional roles of *M. lutarioriparius* GT43 family genes are conserved and diversified in xylan biosynthesis.

## Background

Plant cell walls are complex and dynamic structures composed mainly of polysaccharides (cellulose, hemicellulose and pectin), phenolic compounds (lignin) and glycoproteins [1]. Xylans are the major hemicellulosic saccharides in the primary cell walls of grasses and the secondary cell walls of grasses and dicots, ranking as the second most abundant polysaccharides in nature [2]. Xylans are mainly composed of a linear backbone of β-(1,4)-linked D-xylosyl residues with various sidechains that vary among different plant species and tissue types [3]. Based on the sidechain substitutions, xylans can generally be classified as (methyl)glucuronoxylan (GX), arabinoxylan (AX), and glucuronoarabinoxylan (GAX) [3]. As the major xylan in dicot plants, GX is usually decorated with α-1,2-linked glucuronic acid (GlcA) or 4-O-methylglucuronic acid (MeGlcA), and acetylated at C-2 or C-3 [3, 4]. AX has α-1,3-linked arabinose (Ara) sidechains, and presents as typical hemicellulose components in starchy endosperm of cereal grains [3]. GAX is the predominant hemicellulose in grass cell walls, and has sidechains of α-1,2 or α-1,3-linked arabinose (Ara) and GlcA residues [3]. In addition, GX in angiosperm and GAX in several gymnosperm species contain a tetrasaccharide sequence [β-D-Xyl-(1,3)-α-L-Rha-(1,2) -α-D-GalA-(1,4)-D-Xyl] at the reducing end [5–7]. However, no such oligosaccharide has yet been identified for xylans in grasses [8, 9]. It is still in controversy whether this oligosaccharide functions as a primer or as a terminator in xylan backbone biosynthesis [10].

Several xylan-related mutants named as *irregular xylem (irx)* due to secondary cell wall deficiencies have been identified in *Arabidopsis* by reverse genetics approaches [11, 12]. Most of these identified genes encode putative glycosyltransferases (GT) that are involved in the biosynthesis of xylan. IRX9/IRX9L and IRX14/IRX14L from GT43 family as well as IRX10/IRX10L from GT47 family are responsible for the biosynthesis of xylan backbone [13–19]. *IRX9*, *IRX10* and *IRX14* play dominant roles in xylan backbone biosynthesis, and mutations in each gene lead to reduced xylan content and growth defect. By contrast, *IRX9L*, *IRX10L* and *IRX14L* seem to perform partially redundant roles together with their close homologues, as loss-function of these genes have no observable phenotypes and they only partially complement the phenotypes of *irx9*, *irx10* and *irx14* mutants. In addition, double mutations in each gene pairs dramatically enhance the phenotypes of the single mutant [13, 14, 18, 19]. However, a recent study proposed that these gene pairs play equivalent roles in xylan biosynthesis [20]. Furthermore, two members of DUF579 domain-containing proteins, IRX15 and IRX15L, are essential for the normal elongation of xylan backbone [21, 22]. IRX7/IRX7L from GT47 family, IRX8 and PARVUS from GT8 family are required for the biosynthesis of the reducing end oligosaccharide [5, 23–26]. Mutations in these genes lead to almost entirely loss of the tetrasaccharide accompanied with reduced xylan contents, while the xylan backbone elongation activity is not disturbed [5, 23–26].

Recently biochemical and genetic studies have also led to the identification of several genes that are required for the sidechain modifications of xylan. For instance, GLUCURONIC ACID SUBSTITUTION OF XYLAN (GUX) 1, GUX2, GUX4 and GUX5 from GT8 family are proposed to catalyze the addition of GlcA and MeGlcA sidechains to GX backbone [20, 27–29]. GLUCURONOXYLAN METHYLTRANSFERASE (GXMT) 1, a DUF579 domain protein, has been revealed to be responsible for the 4-O-methylation of GlcA residues in GX [30]. In addition, ESKIMO1/TRICHOME BIREFRINGENCE-LIKE (TBL) 29, a DUF231 domain protein, is required for the O-acetylation of xylan backbone [31, 32]. Moreover, several XYLAN ARABINOSYLTRANSFERASE (XAT), members of GT61 family proteins from rice and wheat, are responsible for transferring the Ara residues onto xylan backbone [33, 34]. XYLOSYL ARABINOSYL SUBSTITUTION OF XYLAN (XAX) 1, another member from GT61 family in rice, is involved in transferring the Xyl residues in β-Xyl*p*-(1 → 2)-α-Ara*f* -(1 → 3) sidechain [34].

Grass xylans have several unique features compared to those from dicots. GX is the most abundant hemicellulose in dicots, while grass xylans usually contain many Ara residue substitutions and thus are termed as GAX or AX [3]. Even though there are clear differences in xylan structure between grasses and dicots, accumulating evidence implicates that GT43 members are functionally conserved in xylan biosynthesis between dicots and monocots. For example, four rice *IRX9* orthologues *OsGT43A*, *OsGT43C*, *OsGT43E* and *OsGT43F* can fully or partially rescue the xylan defect phenotype of *irx9*, while *OsGT43J* is able to complement the xylan defect phenotype of *irx14* in *Arabidopsis* [35, 36]. Three poplar *IRX9* orthologues *PtrGT43A*, *PtrGT43B* and *PtrGT43E* are capable of rescuing the defects of *irx9*, whereas the other two *IRX14* orthologues *PtrGT43C* and *PtrGT43D* are able to complement the phenotypes of *irx14* [37]. Furthermore, it has been demonstrated that rice and poplar GT43 family proteins are evolved to retain two functionally non-redundant groups involved in xylan backbone biosynthesis [36–38]. Additionally, two GT43 members *GhGT43A1* and *GhGT43C1* from cotton have been revealed to be functional orthologues of *Arabidopsis IRX9* and *IRX14*, respectively, and have been shown to participate in xylan backbone biosynthesis during fiber development [39].

*Miscanthus* is a perennial rhizomatous grass with superior characteristics as a bioenergy plant such as high photosynthetic efficiency, low fertilizer and water demand, wide adaptability and high biomass yield. It has attracted increasing attention and concern worldwide as an ideal lignocellulosic feedstock for next-generation bioenergy production [40–42]. Hemicelluloses account for 29–42 % of the *Miscanthus* cell walls [43], and the most abundant hemicellulosic polysaccharide is AX [43, 44], which is also the typical xylan in grass cell walls [45]. It has been shown that hemicellulose exerts dominant and positive effects on biomass digestibility by affecting cellulose crystallinity after pre-treatment with alkali or acid [46]. Although much progress has been gained in the understanding of xylan biosynthesis in the model plant *Arabidopsis thaliana*, relatively less is known about xylan biosynthesis in grasses. To the best of our knowledge, none of GTs responsible for the biosynthesis of xylan has been isolated and characterized in *Miscanthus* as yet.

To provide insight into xylan biosynthesis in *Miscanthus*, we identified seven *GT43* genes in *M. lutarioriparius* and characterized their functional roles in xylan biosynthesis. Complementation assay including plant height, irregular xylem cells in stem cross sections and xylose content measurements revealed that *MlGT43* genes have evolved into two distinct functional groups, in which *MlGT43A-E* are orthologous to *IRX9*, while *MlGT43F* and *MlGT43G* are orthologous to *IRX14*. Furthermore, our results indicated that substantial divergence has occurred in the functional roles of *MlGT43s* during xylan biosynthesis especially in seed coat mucilage. The results presented deepened our understanding of xylan biosynthesis in grasses and may lay the foundation for future genetic manipulation of *Miscanthus* cell wall structure and components.

## Results

### Isolation of *GT43* genes in *M. lutarioriparius*

**Fig. 1:**
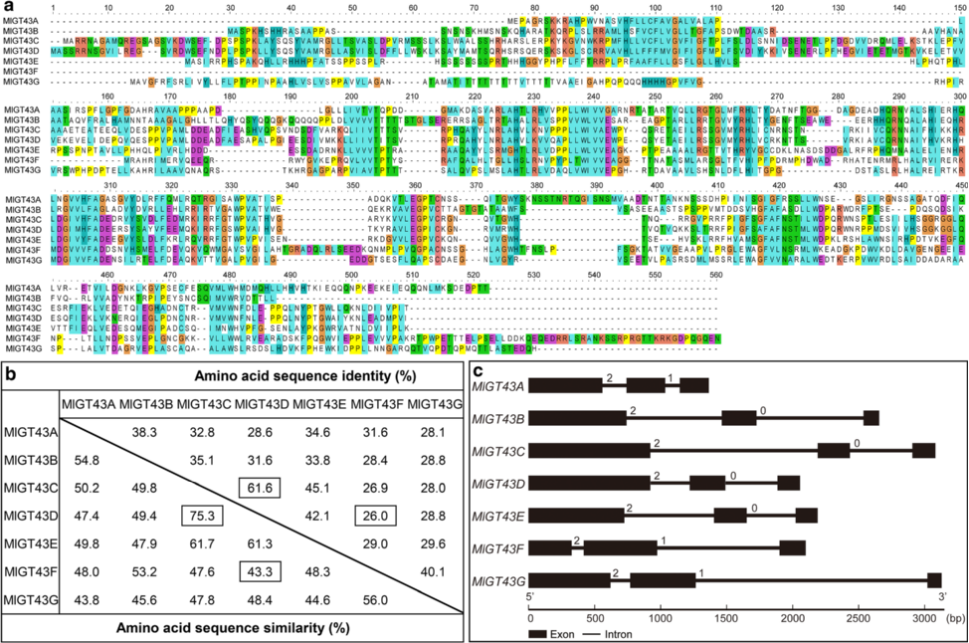
Sequence alignment, identities and gene structure of *MlGT43*. **a** Sequence alignment of seven MlGT43 proteins. **b** Sequence identities and similarities among MlGT43 proteins. The highest and lowest in sequence identity and similarity are outlined. **c** Gene structure of *MlGT43* genes. Exons and introns are represented by filled boxes and lines, respectively. The sizes of exons and introns are proportional to the scale at bottom

To identify the GT43 family in *M. lutarioriparius*, the amino acid sequences of four *Arabidopsis* GT43 members were used as query baits to BLAST against the draft genome sequences of *M. lutarioriparius*, and seven *GT43* orthologous genes were identified. Specific primers were designed and seven candidate genes encoding putative GT43 proteins designated as *MlGT43A* to *MlGT43G* were obtained by PCR in *M. lutarioriparius*. As indicated in Fig. 1a, all seven proteins had a conserved structure and ranged in size from 358 to 451 amino acids. Pairwise comparison of the amino acid sequences showed that MlGT43C and MlGT43D shared the highest sequence similarity (75.3 %), while MlGT43D and MlGT43G shared the lowest sequence similarity (43.3 %) (Fig. 1b).

Deduced MlGT43A and MlGT43B amino acid sequences shared the highest sequence identities with *Arabidopsis* IRX9 (37 and 41 %), and MlGT43C-E shared relatively higher sequence identities with IRX9L (42, 48 and 53 %) than with IRX14 or IRX14L. By contrast, MlGT43F and MlGT43G proteins had the highest sequence identities with IRX14 and IRX14L (59 and 37 %) than with IRX9 (Additional file 1: Table S1).

Furthermore, the gene structure of each *MlGT43* was obtained through the alignment of their coding sequences and genomic sequences (Fig. 1c). All *MlGT43* genes shared very similar gene structure in terms of intron number and exon length. They all contained three exons and two introns. In addition, the intron phases with respect to codons were well conserved among different *MlGT43* genes.

### Phylogenetic analysis of GT43 members from *M. lutarioriparius* and other plant species

**Fig. 2:**
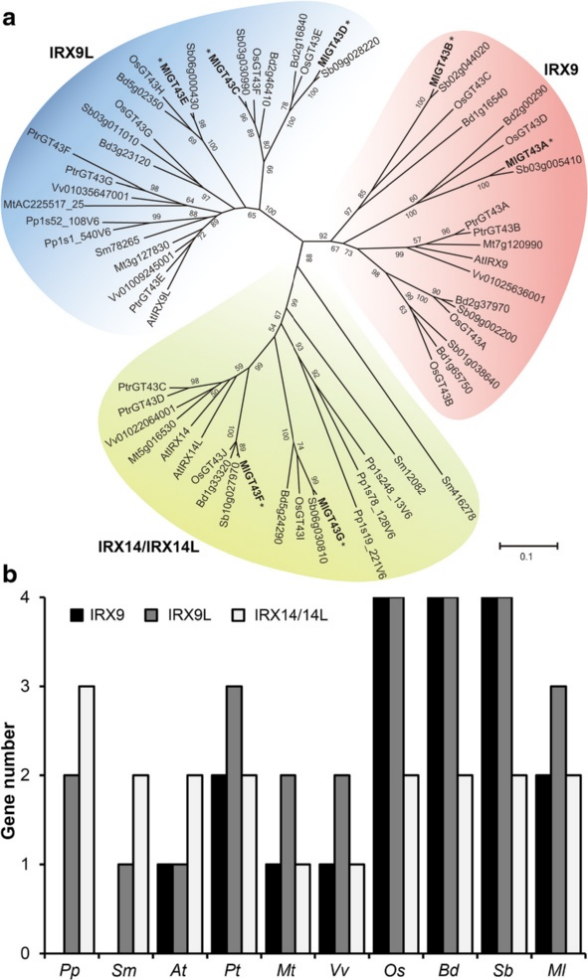
Phylogenetic analysis of GT43 family from *Miscanthus* and nine other plant species. **a** Phylogenetic tree of 64 GT43 proteins from ten plant species. The sequences of 64 GT43 proteins were aligned using ClustalW and their phylogenetic relationship was analyzed using the Neighbor-Joining method in MEGA 6.0. Numbers at nodes indicate the percentage bootstrap scores and only bootstrap values higher than 50% from 1,000 replicates are shown. MlGT43 proteins are marked with asterisks. **b** Distribution of the GT43 proteins from selected plant lineages. *Pp, Physcomitrella patens; Sm, Selaginella moellendorffii; At, Arabidopsis thaliana; Pt, Populus trichocarpa; Mt, Medicago truncatula; Vv, Vitis vinifera; Os, Oryza sativa; Bd, Brachypodium distachyon; Sb, Sorghum bicolor; Ml, Miscanthus lutarioriparius*

To gain insight into the origin and evolutionary history of the GT43 family, we further identified GT43 proteins from nine other currently sequenced genomes that cover a wide spectrum of plant taxonomic groups including moss (*Physcomitrella patens*), spikemoss (*Selaginella moellendorffii*), the monocot angiosperms (*Oryza sativa*, *Brachypodium distachyon* and *Sorghum bicolor*), and the dicot angiosperms (*Arabidopsis thaliana*, *Populus trichocarpa*, *Medicago truncatula* and *Vitis vinifera*). Totally 57 GT43 proteins were identified from these nine plant species (Additional file 2) and a phylogenetic tree was constructed with these GT43 proteins (Fig. 2a). The phylogenetic tree separated all GT43 proteins into three distinct subfamilies designated as IRX9, IRX9L and IRX14/IRX14L, which was similar to the previous studies [13, 38]. The seven GT43 proteins from *Miscanthus* were classified into the three subfamilies. MlGT43A and MlGT43B were clustered into the IRX9 subfamily, MlGT43C-E were classified into the IRX9L subfamily, while MlGT43F and MlGT43G were distributed into the IRX14/IRX14L subfamily.

The distribution of the three subgroups among the ten plant species varied within each subfamily (Fig. 2b). It is noteworthy that the number of GT43 proteins in the monocot species seems to be higher than that of the dicot species, at least it is the case for the selected plant species. For example, there were 10, 10, 10 and 7 members in the monocot species *O. sativa*, *B. distachyon*, *S. bicolor* and *M. lutarioriparius,* whereas the number of GT43 in the dicot species *A. thaliana*, *P. trichocarpa*, *M. truncatula* and *V. vinifera* were 4, 7, 4 and 4, respectively. In addition, the members of IRX9 and IRX9L subfamilies in the monocot angiosperms were generally higher than those of the dicot species. For instance, the IRX9 subfamily accounted for 40, 40, 40 and 28 % in the monocot species *O. sativa*, *B. distachyon*, *S. bicolor* and *M. lutarioriparius*, respectively, whereas the percentages of the IRX9 subfamily in the dicot species *A. thaliana*, *P. trichocarpa*, *M. truncatula* and *V. vinifera* were 25, 25, 28 and 25 %, respectively. Noticeably, no IRX9 subfamily members were present in *P. patens* and *S. moellendorffii.*

### *MlGT43* genes are ubiquitously expressed and have specific expressions in stem cells

**Fig. 3:**
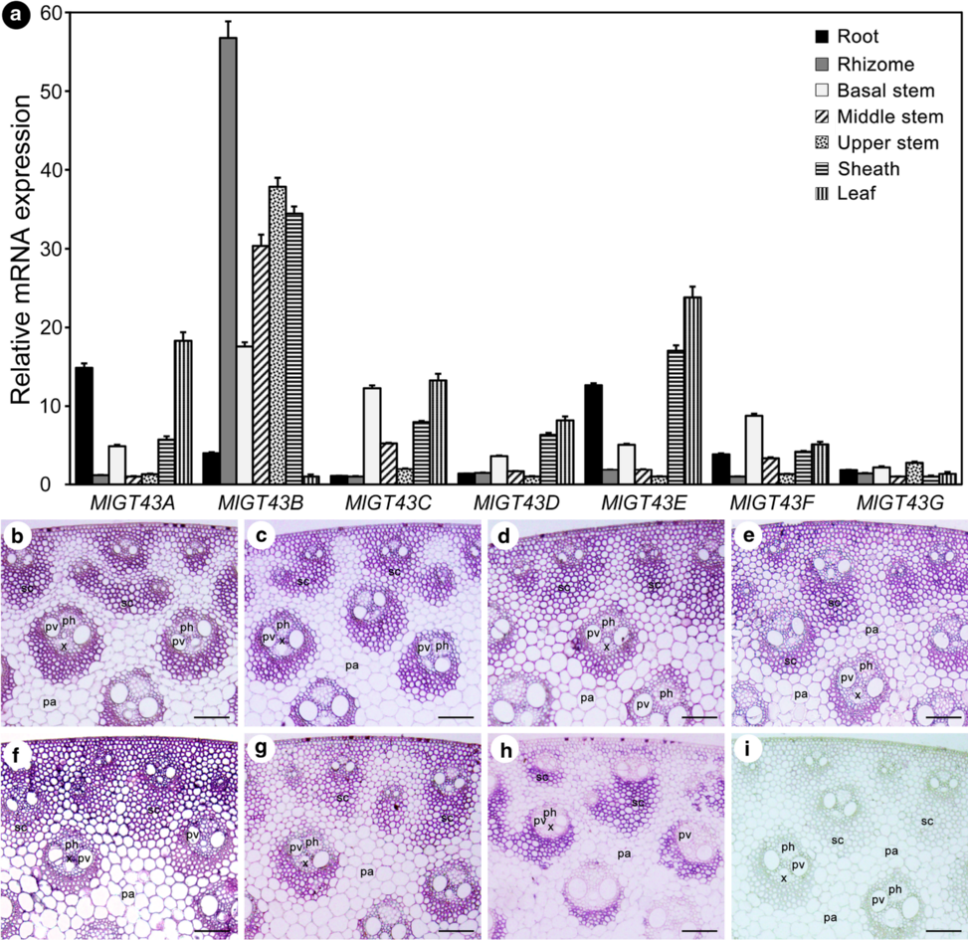
Expression patterns of *MlGT43* genes. **a** Expression analysis of *MlGT43* genes by qRT-PCR. Relative expression levels in seven tissues were normalized using *MlACT11* as the reference gene. For each gene, the tissues with the lowest expression level are set to 1. Data are the means ± SE of three biological replicates. **b** In situ localization of *MlGT43* genes in *Miscanthus* stem. Cross-sections of stems were hybridized with digoxigenin-labeled antisense *MlGT43A* (**b**)*, MlGT43B* (**c**)*, MlGT43C* (**d**), *MlGT43D* (**e**)*, MlGT43E* (**f**), *MlGT43F* (**g**), *MlGT43G* (**h**), or sense (**i**) RNA probes, and the hybridization signals were detected with alkaline phosphatase-conjugated antibody and were shown as purple color. pv, pitted vessel; x, xylem; ph, phloem; pa, parenchyma; sc, sclerenchyma. Bar = 100 μm

To investigate the expression patterns of *MlGT43* genes, we first used the quantitative real-time RT-PCR (qRT-PCR) to examine their expressions across seven different tissues. As shown in Fig. 3a, all seven *MlGT43* genes were ubiquitously expressed in seven different tissues examined, but their relative expression levels differed significantly. For example, *MlGT43A*, *MlGT43D* and *MlGT43E* genes shared similar expression patterns with predominant expressions in leaf, whereas the expressions of *MlGT43B* and *MlGT43G* genes were relatively lower. *MlGT43C* and *MlGT43F* genes were broadly expressed in the majority of the tissues, while especially higher expressions were detected in the basal stem. Furthermore, all *MlGT43* genes except *MlGT43B* exhibited higher expressions in the basal stem than in the upper stem.

To obtain more detailed expression patterns of *MlGT43* genes in specific cell types, we further performed the in situ hybridization analysis to examine their expressions in the 11^th^ internode of the stem. For all seven genes, intense hybridization signals were observed in sclerenchyma cells and vascular bundle fiber cells, the cell types undergoing secondary wall thickening (Fig. 3b-h). Moreover, relatively weak hybridization signals were also observed for *MlGT43C-E* in parenchyma cells. By contrast, the control hybridized with sense probes did not show any signals in vascular bundle or sclerenchyma cells (Fig. 3g). These results suggest that *MlGT43* genes may participate in diverse plant development processes especially in the secondary cell wall formation.

### MlGT43 members are targeted to Golgi apparatus

**Fig. 4:**
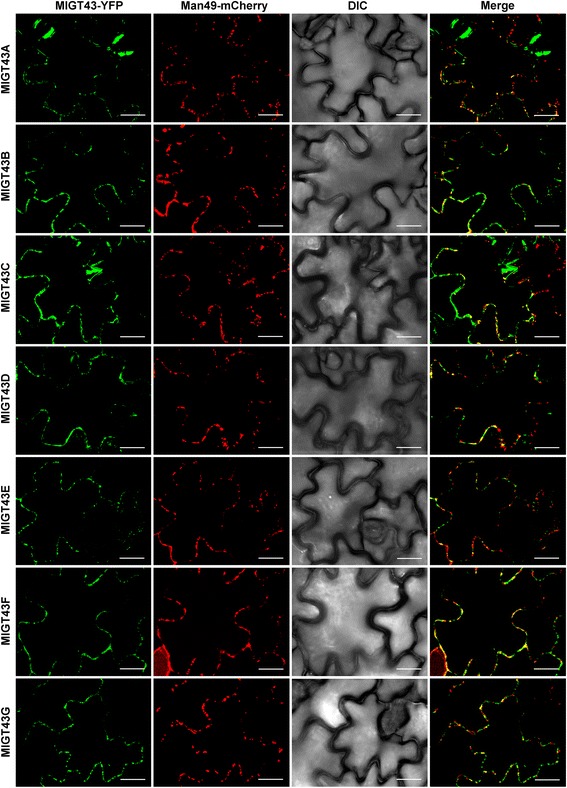
Subcellular localization of YFP-tagged MlGT43 proteins. YFP-tagged MlGT43 proteins were transiently expressed in leaf epidermal cells of *Nicotiana benthamiana*, and their subcellular locations were examined with a laser scanning confocal microscope. The single-plane confocal micrographs of MlGT43 proteins fused with C-terminal YFP, the Golgi marker Man49-mCherry, differential interference contrast (DIC) image, and merged YFP and mCherry channels are shown. Note the superimposition of YFP-MlGT43s and Man49-mCherry signals. Bar = 20 μm

To investigate the subcellular localization of MlGT43 proteins, we constructed fluorescently tagged fusion proteins by fusing Yellow Fluorescent Protein (YFP) to the C terminus of each MlGT43 protein. The recombinant constructs were transiently co-expressed in *Nicotiana benthamian*a leaf epidermal cells with the Golgi marker Man49-mCherry [47]. Examination of the fluorescent signals revealed that seven YFP-tagged MlGT43s all exhibited a punctate distribution, and the pattern perfectly matched with that of Man49-mCherry (Fig. 4), whereas the YFP control protein had signals throughout the cytoplasm and the nucleus (data not shown). The co-localization of MlGT43 proteins with the Golgi marker indicate that MlGT43s are Golgi-localized proteins.

### *MlGT43* genes rescue the morphological defects of *irx9* or *irx14*

**Fig. 5:**
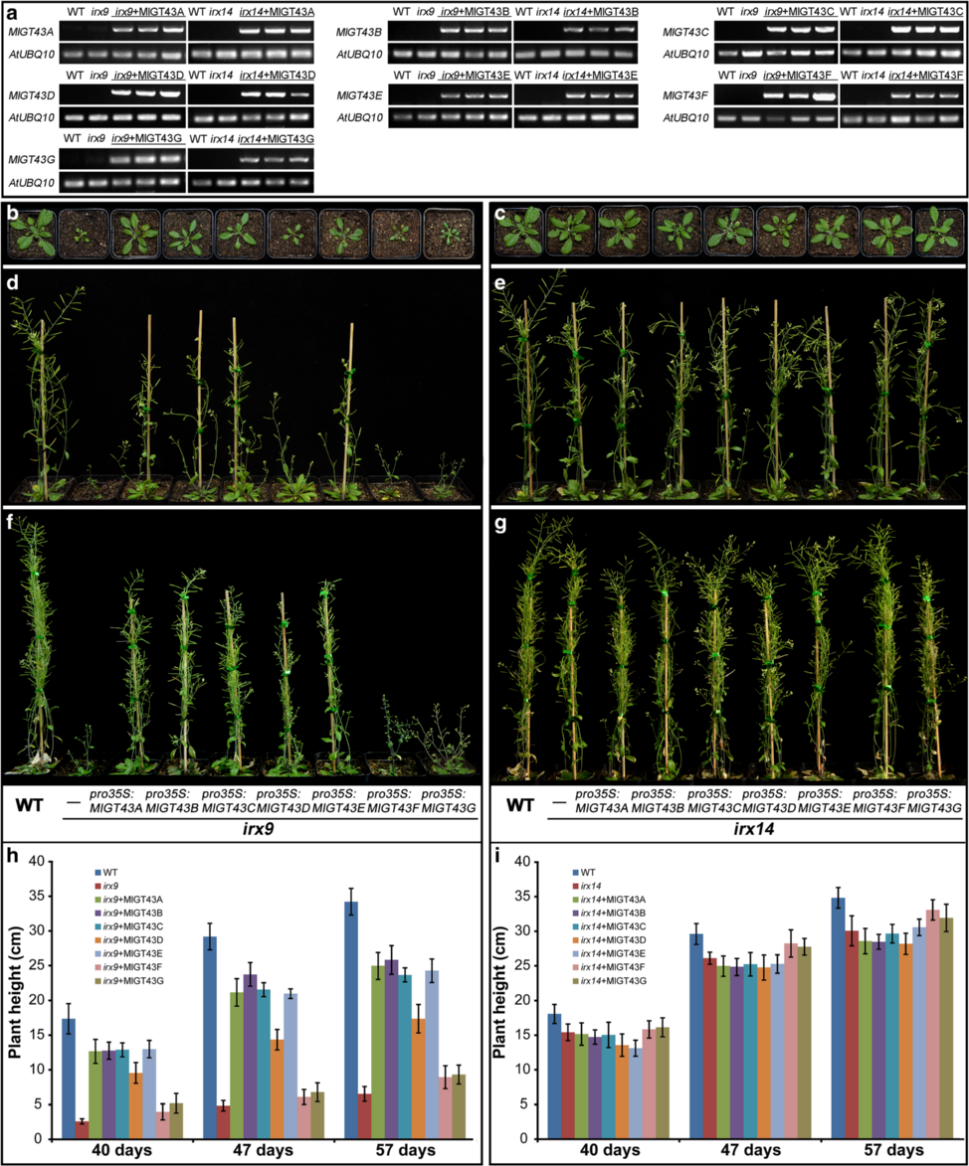
Expression of seven *MlGT43* genes in *Arabidopsis irx9* or *irx14* mutants. **a** RT-PCR detection of the *MlGT43* transcripts in the complemented *irx9* or *irx14* plants. The *Arabidopsis UBQ10* gene was used as a reference. **b**, **d**, **f** Phenotype of four-, six- and eight-week-old soil-grown WT, *irx9* and *MlGT43s* complemented *irx9* plants. **c**, **e**, **g** Phenotype of four-, six- and eight-week-old soil-grown WT, *irx14* and *MlGT43s* complemented *irx14* plants. **h** Stem height of the WT, *irx9* and *MlGT43s* complemented *irx9* plants through 40, 47, 57 days of growth. **i** Stem height of the WT, *irx14* and *MlGT43s* complemented *irx14* plants through 40, 47, 57 days of growth. Data are means ± SD from at least twelve plants for each background. Two homozygous T3 lines of *MlGT43s* complemented *irx9* or *irx14* were used in the analysis

To reveal whether *MlGT43* genes perform the same functions as *IRX9* and *IRX14* orthologues in *Arabidopsis*, we examined their abilities to rescue the morphological defects of *irx9* and *irx14*. Due to the severely dwarfed plant stature and poor fertility of homozygous *irx9* plants [5], we used the heterozygous line for the transformation with the 35S:*MlGT43*s constructs. Positive transgenic lines for each construct were tested for the presence of *MlGT43* genes in homozygous *irx9* and *irx14* background by semi-quantitative RT-PCR (Fig. 5a). Homozygous T2 plants from at least two independent transformants with higher expressions were used for the phenotypic analyses.

The growth of the *irx9* plants was characterized by the dwarf stature, smaller rosette size and dark-green leaves under our growth conditions, which is similar to the previous reports [5, 12]. Overexpression of *MlGT43A-E* genes in *irx9* displayed an intermediate growth phenotype between the mutant and the wild type (WT) in terms of rosette size and inflorescence height. The rosette diameters of the complemented plants increased by two- to three-fold, and the inflorescence stems were two- to four-fold taller compared to the *irx9* plants after four weeks of growth (Fig. 4b, d), suggesting that the *irx* phenotype may be partially complemented in these transformants. By contrast, transformants of *MlGT43F* or *MlGT43G* overexpression in *irx9* mutant exhibited a morphology resembled of the *irx9* mutant, indicating that *MlGT43F* and *MlGT43G* were unable to complement the *irx9* phenotypes (Fig. 4b, d, f).

The growth of *irx14* mutant did not show any other obvious phenotypes except for a slight reduction in plant height compared to WT (Fig. 4c, e) as described previously [14]. The height of all *MlGT43* complemented *irx14* plants was indistinguishable from that of *irx14* or WT plants, thus it is hard to evaluate the ability of seven *MlGT43* genes to complement the *irx14* mutant merely judged from their growth phenotypes. Subsequently, xylem morphology, xylan immunolocalization and cell wall monosaccharide compositions will be further examined in the transgenic plants to determine the abilities of *MlGT43s* to complement the *irx14* phenotypes.

### Microscopic analysis of the secondary cell wall

**Fig. 6:**
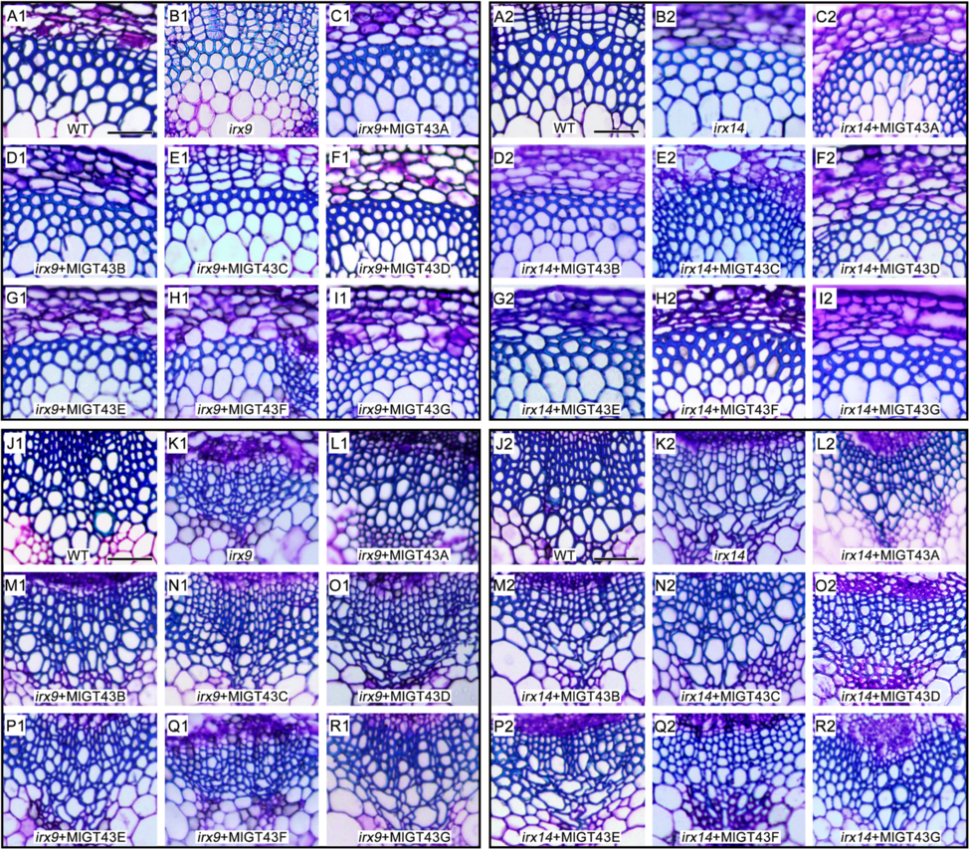
Morphology of xylem and interfascicular fibers of WT, *irx9*, *irx14* and *MlGT43* complemented plants. Stems of eight-week-old plants were sectioned (8 μm-thick) and stained with TBO for examination of the morphology of vessels, xylary fibers and interfascicular fibers. **A1**-**I1**, interfasicular fibers for WT, *irx9* and *MlGT43* complemented *irx9* plants. **A2**-**I2**, interfasicular fibers for WT, *irx14* and *MlGT43* complemented *irx14* plants. **J1**-**R1**, xylary fibers and vessels for WT, *irx9* and *MlGT43* complemented *irx9* plants. **J2**-**R2**, xylary fibers and vessels for WT, *irx14* and *MlGT43* complemented *irx14* plants. At least two homozygous T3 lines of *MlGT43s* complemented *irx9* or *irx14* were used in the analysis. Images for each tissue are set as the same magnification. Bar = 50 μm

To demonstrate whether the morphological complementation by *MlGT43* genes could be accompanied with the rescue of xylem morphology, the basal inflorescence stems of each complemented line were sectioned and observed by light and transmission electron microscopy. Toluidine blue O (TBO) staining was performed on stem sections of WT, *irx9*, *irx14* and complemented plants to examine the morphology of secondary cell walls. As shown in Fig. 6, all *MlGT43A-E* complemented *irx9* plants exhibited dramatically thickened cell walls in interfascicular fibers compared to *irx9*. The majority of xylem vessels in *MlGT43A* and *MlGT43B* complemented *irx9* plants were characterized by large open round cells comparable to those in WT plants (Fig. 6C1, D1, L1, M1). In addition, the xylem vessels of *MlGT43C*, *MlGT43D* or *MlGT43E* complemented *irx9* plants were usually smaller in size with occasionally irregular shapes, probably due to the not fully thickened cell walls compared to WT (Fig. 6 E1-G1, N1-P1). By contrast, overexpression of *MlGT43F* or *MlGT43G* in *irx9* could not restore the collapsed vessels and the weakly thickened interfascicular fibers in *irx9* (Fig. 6 H1, I1, Q1, R1), which is in consistency with their growth phenotypes (Fig. 5b, d).

The homozygous *irx14* plants also showed collapsed xylem vessels and thinner secondary cell walls, which is consistent with the previous study [15]. Overexpression of either *MlGT43F* or *MlGT43G* could almost fully rescue the *irx* phenotype of *irx14* as witnessed by a relatively less irregular vessel cells compared to *irx14*. However, the complemented lines still retained relatively thinner cell walls in both interfascicular fibers and xylem vessels compared to WT (Fig. 6 H2, I2, Q2, R2). By contrast, overexpression of *MlGT43A-E* in *irx14* displayed a collapsed xylem vessel and thinner fiber cell wall phenotype that was indistinguishable from the *irx14* mutant (Fig. 6 C2-G2, L2-P2), indicating that *MlGT43A-E* genes could not rescue the defects of *irx14*.

**Fig. 7:**
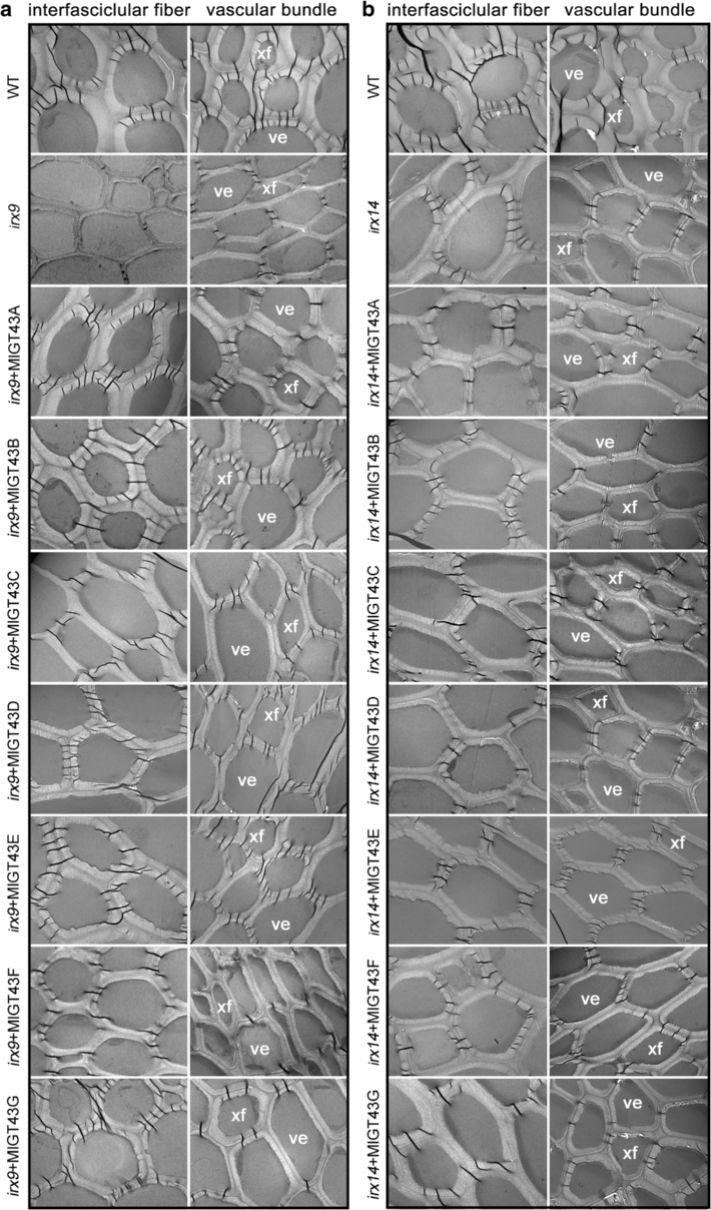
Transmission electron micrographs of stem sections of WT, *irx9*, *irx14* and *MlGT43* complemented plants. Stems of eight-week-old plants were cut into 70 nm-thick sections and observed with transmission electron microscope, indicating increased fiber and vessel wall thickness by expression of *MlGT43* genes. **a**, Transmission electron micrographs of stem sections of *MlGT43* complemented *irx9* lines. **b**, Transmission electron micrographs of stem sections of *MlGT43* complemented *irx14 *lines. At least two homozygous lines of MlGT43 complemented *irx9* or *irx14* were used in the analysis. ve, vessels, xf, xylary fibers. Bar = 5 μm

Transmission electron microscopy confirmed that the thickness of interfascicular fiber cell walls of the *MlGT43A-E* complemented *irx9* plants was intermediate between *irx9* and WT (Fig. 7a and Table 1). Meanwhile, the wall thickness of xylary fibers and vessels in *MlGT43A-E* complemented *irx9* lines was also significantly increased but not restored to the WT level. By contrast, the wall thickness of interfascicular fibers, xylary fibers and vessels of *MlGT43F* or *MlGT43G* complemented *irx9* plants was similar to that of the *irx9* mutant (Fig. 7a and Table 1). The wall thickness of interfascicular fibers, xylary fibers and vessels for *MlGT43F* or *MlGT43G* complemented *irx14* plants was intermediate between *irx14* and WT, while the wall thickness for *MlGT43A-E* complemented *irx14* lines was similar to that of *irx14* (Fig. 7b and Table 1). Together, these results indicate that *MlGT43A-E* can fully or partially rescue the *irx9* but not the *irx14* phenotypes, while *MlGT43F* and *MlGT43G* can complement the *irx14* but not the *irx9* defects.

**Table 1:** Cell wall thickness of fiber and vessel cells in the stems of WT, *irx9*, *irx14*, and *MlGT43s* complemented plants

	Interfascicular fiber (μm)	Vessel (μm)	Xylary fiber (μm)
WT	1.98 ± 0.11	1.35 ± 0.26	1.46 ± 0.28
*irx9*	1.15 ± 0.23	0.47 ± 0.10	0.59 ± 0.18
*irx9 +* MlGT43A	1.66 ± 0.19	1.21 ± 0.10	1.23 ± 0.10
*irx9 +* MlGT43B	1.68 ± 0.33	1.19 ± 0.14	1.23 ± 0.20
*irx9 +* MlGT43C	1.62 ± 0.25	0.97 ± 0.05	1.07 ± 0.15
*irx9 +* MlGT43D	1.36 ± 0.29	0.90 ± 0.08	0.97 ± 0.20
*irx9 +* MlGT43E	1.40 ± 0.18	0.95 ± 0.19	0.93 ± 0.14
*irx9 +* MlGT43F	1.26 ± 0.18	0.62 ± 0.14	0.63 ± 0.17
*irx9 +* MlGT43G	1.23 ± 0.26	0.59 ± 0.12	0.65 ± 0.11
*irx14*	1.49 ± 0.25	0.98 ± 0.08	1.01 ± 0.22
*irx14 +* MlGT43A	1.46 ± 0.30	0.97 ± 0.07	1.00 ± 0.19
*irx14 +* MlGT43B	1.47 ± 0.19	0.93 ± 0.30	0.95 ± 0.10
*irx14 +* MlGT43C	1.50 ± 0.13	0.96 ± 0.11	0.96 ± 0.15
*irx14 +* MlGT43D	1.46 ± 0.24	0.95 ± 0.13	0.97 ± 0.17
*irx14 +* MlGT43E	1.48 ± 0.21	0.97 ± 0.14	0.99 ± 0.13
*irx14 +* MlGT43F	1.53 ± 0.13	1.04 ± 0.16	1.12 ± 0.12
*irx14 +* MlGT43G	1.58 ± 0.11	1.10 ± 0.17	1.20 ± 0.11

At least two independent transgenic lines for each construct were used for measurement. WT, *irx9*, and *irx14* were included for comparison. Eight-week-old plants for each background were used for analysis. Wall thickness was measured from transmission electron micrographs of fibers and vessels. Data are means (μm) ± SE from 20 cells

### Immunolocalization of xylan in *MlGT43s* complemented lines

**Fig. 8:**
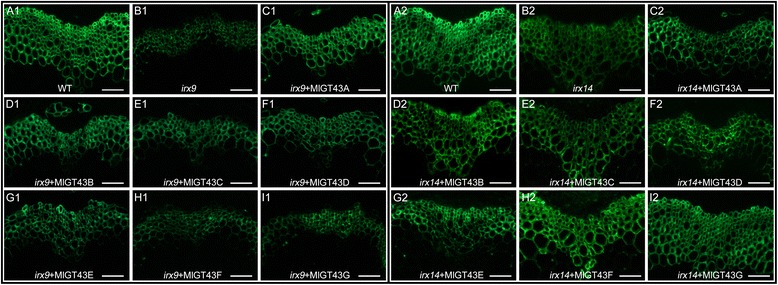
Immunolocalization of xylan using the monoclonal antibody LM10. Labelling was carried out on 8 μm-thick transverse sections from stem tissues of eight-week-old plants. A1-I1: xylan immumolocalization in WT, *irx9* and *MlGT43* complemented *irx9* lines. A2-T2: xylan immunolocalization in WT, *irx14* and *MlGT43* complemented *irx14* lines. Signals were detected with Alexa Fluor488-conjugated secondary antibody and observed with a BX51 fluorescence microscope (OLYMPUS). Bar = 50 μm

To investigate whether the phenotypes of the complemented plants are correlated with xylan deposition in secondary cell walls, we performed immunolocalization of xylan using the xylan-directed monoclonal antibody LM10, which recognizes unsubstituted or low-substituted xylan [48], to examine the distribution of xylan in the cell walls. As indicated in Fig. 8, strong fluorescence signals were present in the cell walls of interfascicular fibers and xylem cells in the WT stem, however, relatively weaker signals were detected in the corresponding tissues of the *irx9* plants, although the overall pattern of labeling was unchanged compared with the WT plants (Fig. 8 A1, B1). In *MlGT43A* and *MlGT43B* complemented *irx9* lines, the intensity of fluorescence signals was almost restored to the WT level, and the overall pattern of labeling was almost identical to that of WT, indicating that the GX content in interfascicular fibers and xylem cells was nearly restored to the WT level (Fig. 8 C1, D1). The LM10 signals in the *MlGT43C-E* complemented *irx9* plants were intermediate between *irx9* and WT plants (Fig. 8 E1-G1). By contrast, the LM10 signals for *MlGT43F* and *MlGT43G* complemented *irx9* lines were relatively weaker compared with the others, and the intensity was comparable to that of the *irx9* mutant (Fig. 8 H1, I1). As for the *irx14* background, the intensity of fluorescence signals of *MlGT43F* and *MlGT43G* complemented lines was comparable to that of WT in xylem cells and interfascicular fibers (Fig. 8 H2, I2). By contrast, *MlGT43A-E* complemented *irx14* lines exhibited nearly equal signal intensity to the *irx14* mutant (Fig. 8 C2-G2). These results indicate that MlGT43A-E perform a similar biochemical function as IRX9, whereas MlGT43F and MlGT43G share a conserved biochemical function with IRX14, thus leading to a restoration of normal xylan synthesis in their complemented plants.

### Analysis of cell wall composition

**Fig. 9:**
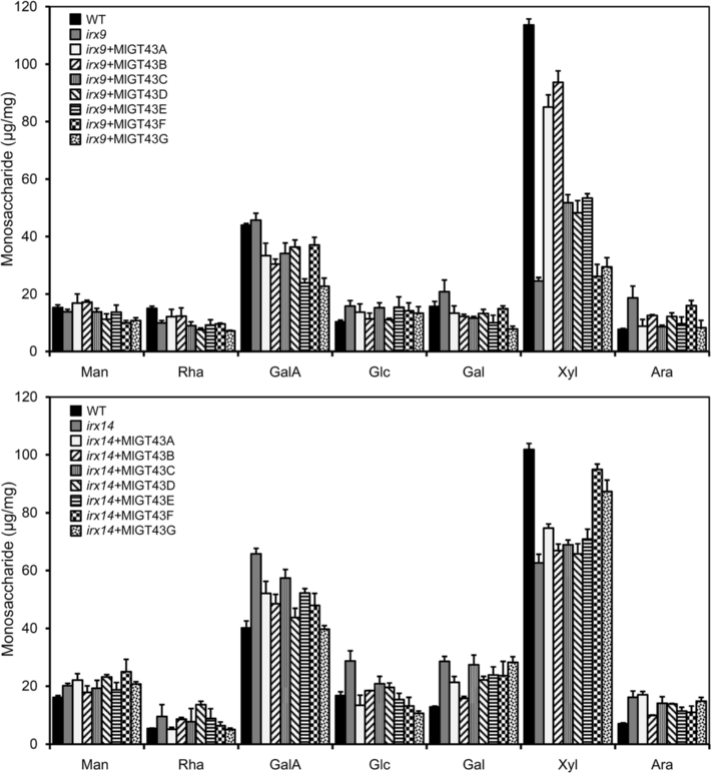
Monosaccharide composition of cell walls isolated from the stems of WT, *irx9*, *irx14* and *MlGT43* complemented plants. Cell walls were prepared from inflorescence stems of eight-week-old plants and their glycosyl compositions were determined by HPLC. Data are means ± SD of three independent analyses

To determine whether the complementation of xylem morphology and xylan deposition is correlated with the restoration of chemical composition, we measured the monosaccharide composition, cellulose and lignin contents of the transgenic lines. Monosaccharide composition analysis was performed on cell wall preparations from eight-week-old inflorescence stems of WT, *irx9*, *irx14* and *MlGT43* complemented lines (Fig. 9). The xyl content in *irx14* was decreased by 40 % compared to WT, whereas it was decreased more dramatically in *irx9*, with only 21 % of the WT. The transgenic plants overexpressing *MlGT43A* and *MlGT43B* in *irx9* significantly increased the content of xyl to 73 and 82 % of the WT level, respectively. A modest increase was also observed in the *MlGT43C-E* complemented *irx9* lines. However, no significant increases in xyl content were observed in *MlGT43F* or *MlGT43G* complemented *irx9* lines compared to *irx9*. Overexpression of *MlGT43F* and *MlGT43G* in *irx14* restored the xyl content to 92 and 83 % of the WT, respectively. The xyl content of *MlGT43A*-*E* complemented *irx14* plants was individually increased by approximately 5 to 10 % compared to *irx14*.

In addition, mutations of *irx9* and *irx14* caused significant reductions in cellulose and lignin contents compared to WT. Not unexpectedly, overexpression of *MlGT43A-E* but not *MlGT43F* and *MlGT43G* in *irx9* restored the contents of cellulose and lignin almost to the WT level. Similarly, overexpression of *MlGT43F* and *MlGT43G* but not *MlGT43A-E* in *irx14* recovered the levels of cellulose and lignin nearly to the WT level (Additional file 3: Figure S1). These results further indicate that *MlGT43A-E* but not *MlGT43F-G* can partially restore the xylan biosynthesis in *irx9*, while *MlGT43F*-*G* but not *MlGT43A-E* are able to rescue the xylan biosynthesis defect in *irx14*, suggesting that *MlGT43A-E* are orthologous to *IRX9*, while *MlGT43F* and *MlGT43G* are orthologous to *IRX14.*

### Transactivation assay for *MlGT43* genes

**Fig. 10:**
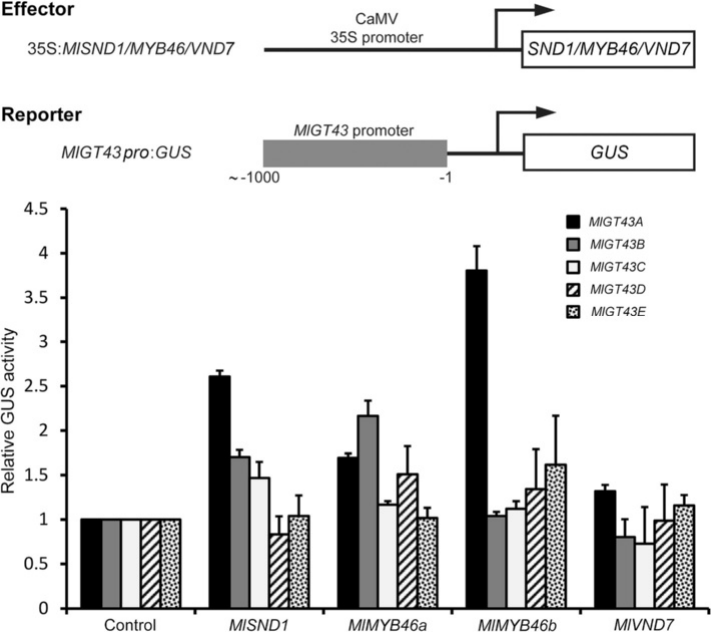
Transactivation assay of the *MlGT43A-E* promoters by *MlSND1*, *MlMYB46a/b* or *MlVND7*. Diagrams indicate the effector and reporter constructs used for transactivation analysis. The effector constructs contain the *MlSND1*, *MlMYB46a*, *MlMYB46b* or *MlVND7* cDNA driven by the 35S promoter. The reporter constructs consist of the GUS reporter gene driven by the *MlGT43A-E* promoters. Transactivation ability was represented by the relative GUS activities. The expression level of the GUS reporter gene in *Arabidopsis* leaf protoplasts transfected with no effector was used as a control and was set to 1

*SND1* (*SECONDARY WALL-ASSOCIATED NAC DOMAIN PROTEIN 1*), *VND7* (*VASCULAR-RELATED NAC-DOMAIN 7*) and *MYB46* have been shown to act as the master switches in the regulatory network of secondary cell wall biosynthesis [49]. To better understand the underlying regulatory mechanism of *MlGT43* genes, we isolated the orthologues of *SND1*, *VND7* and *MYB46* in *M. lutarioriparius* and analyzed their transactivation abilities on pro*MlGT43A-E*:GUS reporters using a transient transactivation assay (Fig. 10). The results showed that *MlGT43A* was transactivated by *MlSND1*, *MlMYB46a*, *MlMYB46b* and *MlVND7. MlGT43B* was also transactivated by *MlSND1*, *MlMYB46a*, but not by *MlMYB46b* and *MlVND7*. By contrast, *MlGT43C-E* were not transactivated by any effectors examined. These results indicate that *MlGT43A* and *MlGT43B* genes are differentially regulated by *SND1*, *MYB46* and *VND7* orthologues and there probably exist other transcriptional factors regulating the expression of *MlGT43C*-*E* genes besides the above effectors examined.

### None of *MlGT43* genes could rescue the mucilage defects of *irx14* seeds

Since *IRX14* has been shown to be responsible for the synthesis of xylan in seed coat mucilage and mutations in *IRX14* lead to a defect in mucilage cohesiveness property [50, 51], we sought to examine whether *MlGT43* genes could rescue the mucilage defect of *irx14*. The seeds of *MlGT43* complemented lines in *irx14* background were examined by ruthenium red staining (Additional file 4: Figure S2). When seeds were imbibed in water and subjected to gentle shaking, the seeds of seven *MlGT43* complemented *irx14* lines all exhibited a thin layer of mucilage phenotype similar to that of the *irx14* seeds. By contrast, the WT seeds have a much thicker mucilage layer tightly attached to the seed. This result indicated that none of *MlGT43* genes could rescue the mucilage defect of *irx14*.

We further determined the monosaccharide composition of seed mucilage for each complemented line. The xyl content was dramatically reduced in *irx14* mucilage as previously reported [50, 51]. Not surprisingly, the xyl content in seven complemented lines was comparable to that of *irx14* and not restored to the WT level (Additional file 5: Figure S3), suggesting that none of MlGT43s could synthesize the xylan in the seed coat mucilage.

## Discussion

Much progress has been gained in xylan biosynthesis mainly in the model species *Arabidopsis*. Several GT43 family proteins have been revealed to participate in xylan backbone biosynthesis in secondary cell walls [13, 19, 35–38]. By contrast, less knowledge regarding the biosynthesis of xylan is known in grass, despite that xylan especially arabinoxylan is the major hemicellulosic components in grass cell walls. In this study, we identified seven *GT43* genes from *M. lutarioriparius* and revealed that they are functional orthologues of *Arabidopsis IRX9* and *IRX14*. Phylogenetic analysis of GT43 proteins from nine representative plant species and *Miscanthus* revealed that these proteins were classified into three major clades, namely IRX9, IRX9L and IRX14/IRX14L (Fig. 2). Noteworthy, our results indicated that no IRX9 orthologues were present in the lower plant species moss (*P. patens*) and spikemoss (*S. mellysellia*). Moss has been demonstrated to be capable of synthesizing glucuronoxylans that are structurally similar to those present in the secondary cell walls of higher plants [52]. The glucuronoxylans are mainly located in primary cell walls in moss as no mechanical supporting tissues composed mainly of secondary cell walls have been evolved. As a basal vascular plant, spikemoss has evolved tissues containing secondary cell walls. Xylans have been shown to be one of the most abundant cell wall components in spikemoss [53]. Since IRX9 has been shown to be mainly responsible for the biosynthesis of xylans in secondary cell walls [13, 19, 20, 35, 38, 54], the absence of xylans in secondary cell walls in moss may partially explain why no IRX9 orthologues are present in moss genome. Thus, it seems likely that vascular plants have evolved a specialized isoform of IRX9, which is responsible for xylan biosynthesis in secondary cell walls. However, this hypothesis seems somewhat implausible because IRX9 orthologues are also lacking in spikemoss. Together, these results indicate that the specialization of IRX9 for xylan biosynthesis in primary and secondary cell walls is not necessary for the evolution of vascular tissue.

Although the qRT-PCR analysis revealed that *MlGT43A* to *MlGT43E* in *M. lutarioriparius* exhibited broad expression patterns across the tissues examined, the in situ hybridization analysis unambiguously indicated that *Miscanthus IRX9* orthologues *MlGT43A* and *MlGT43B* were preferentially expressed in cells undergoing secondary wall thickening, while the *IRX9L* orthologues *MlGT43C-E* were expressed in both parenchymal cells and sclerenchyma cells (Fig. 3). In addition, *IRX9* orthologues *MlGT43A* and *MlGT43B* were both transcriptionally regulated by *MlSND1*, *MlMYB46a* or *MlVND7*, three candidate transcriptional switches governing secondary cell wall biosynthesis. By contrast, the *Miscanthus IRX9L* orthologues (*MlGT43C-E*) were not significantly transactivated by these transcription factors (Fig. 10). Similar results were reported for *IRX9* orthologues in *Arabidopsis*, rice (*OsGT43A* and *OsGT43E*) and poplar (*PtrGT43A* and *PtrGT43B*), which were shown to be highly expressed in tissues with abundant secondary cell walls [13, 35, 38]. In addition, poplar *IRX9* orthologues (*PtrGT43A* and *PtrGT43B*) were transcriptionally regulated by *PtxtMYB021* (*MYB46* orthologue) and *PNAC085* (*SND1* orthologue), master transcriptional switches involved in secondary cell wall formation [38]. Together, these results indicated that *IRX9* orthologues are mainly involved in secondary cell wall biosynthesis, and its roles are highly conserved in angiosperm species.

In addition, the number of GT43 proteins in monocot species seems to be higher than that of dicot species, which was mainly due to a significantly expansion of IRX9 and IRX9L members in monocot species (Fig. 2b). In dicots, such as *Arabidopsis* and poplar, xylan is predominantly deposited in the secondary cell walls, whereas there is very limited amounts of xylan in the primary cell walls. By contrast, the monocot species including rice and *Miscanthus* have abundant amounts of xylan in both primary and secondary cell walls. This could partially explain why the number of IRX9 and IRX9L orthologues are over-presented in monocots compared with dicots.

Phylogenetic analysis also indicated that ancestral *IRX9* orthologues emerged after the specification of the higher plants (Fig. 2a). In addition, *IRX9* may possibly evolve from its *IRX9L* homologue through the duplication events during the evolutionary process as they share very high sequence identities [13, 38]. The functional diversification of *IRX9* orthologues may be due to their expression specificities and their abilities to respond to the key transcriptional factors involved in secondary wall formation (Fig. 10). The different cis-regulatory elements present in the promoter of *Miscanthus IRX9* and *IRX9L* orthologues may explain their functional divergences to some extent (Additional file 6: Table S2). In other words, *Miscanthus IRX9* orthologues may have evolved to gain some key cis-regulatory elements, which confers their specific functions in xylan biosynthesis during secondary cell wall formation.

In *Arabidopsis*, IRX9 and IRX14 play independent roles in xylan biosynthesis, since the phenotypes of *irx9* mutant cannot be rescued by the overexpression of IRX14 or IRX14L and vice versa [13, 19]. In addition, IRX9 and IRX14 are proposed to play dominant roles, whereas their homologues IRX9L and IRX14L are indicated to play partially redundant or minor roles in xylan backbone biosynthesis [13, 14, 19]. Contrary to this assumption, a recent study proposed that IRX9L and IRX14L play equally important roles with IRX9 and IRX14 in xylan biosynthesis [20]. The seven GT43 orthologues in *Miscanthus* were classified into three major subclades namely IRX9, IRX9L and IRX14/IRX14L. All five *Miscanthus* IRX9 and IRX9L orthologues (MlGT43A-E) could nearly fully or partially complement the phenotypes of *irx9*, while none of these genes could rescue the phenotypes of *irx14*. Similarly, two *Miscanthus* IRX14 and IRX14L orthologues (MlGT43F and MlGT43G) were able to rescue the phenotypes of *irx14* but not *irx9*. These results indicated that GT43 genes have been evolved into two functional groups in *Miscanthus*, and the functions between the members in IRX9/IRX9L and IRX14/IRX14L groups have been diversified substantially. Likewise, the involvement of two distinctly functional groups of GT43 genes in xylan biosynthesis seems to be highly conserved in different plant species. For example, the rice orthologues of IRX9 (OsGT43A and OsGT43E) were able to rescue the phenotypes of *irx9* but were not able to complement those of *irx14*. By contrast, the IRX14 orthologue OsGT43J was able to complement the *irx14* phenotypes but unable to rescue those of *irx9*. Similarly, the poplar IRX9 orthologues (PtrGT43A, PtrGT43B and PtrGT43E) were able to rescue the xylan defects of *irx9* but could not complement those of *irx14*, whereas the IRX14 orthologues (PtrGT43C and PtrGT43D) were capable of rescuing the defects of *irx14* but not those of *irx9*.

Xylans are typically substituted with α-l-Ara*f* residues at C2- and/or C3-position in arabinoxylans (AX) and less frequently with GlcpA and/or 4-O-Me-GlcpA sidechains at C2- position in glucuronoarabinoxylans (GAX) in grasses [3, 4]. AX is the major xylan in *Miscanthus* and the degree of Ara*f* substitution positively affects the lignocellulose saccharification under various pretreatments [44, 45]. AX is also the major xylan of the seed mucilage in psyllium (*Plantago ovata*) [55]. During *Arabidopsis* seed differentiation, the seed coat epidermal cells synthesize and secrete large amounts of mucilage, which encapsulated the seed upon imbibition. Although the *Arabidopsis* seed coat mucilage are primarily composed of pectic RG I, minor amounts of xylan are also present in the mucilage and play an important role in maintaining the structure of seed coat mucilage [50, 51]. Unlike the typical xylan in dicot secondary cell walls, mucilage xylan has a unique structure with frequent substitutions with Xyl rather than with GlcA or Ara residues [50, 51]. IRX14 has been revealed to be responsible for the biosynthesis of xylan in *Arabidopsis* mucilage and loss function lead to a mucilage cohesiveness defect [50, 51]. It is noteworthy that none of the *MlGT43* genes could be able to complement the *irx14* mucilage defect (Additional file 4: Figure S2), suggesting that MlGT43s could not synthesize the mucilage xylan, which is involved in maintaining the structure of seed coat mucilage (Additional file 5: Figure S3). The reason might due to the fact that mucilage xylan is structurally different from that of the stem secondary walls, and the functions of *Miscanthus* GT43 proteins have diversified from those of *Arabidopsis* orthologues during the evolutionary process. Similarly, there is also lines of evidence highlighting that mucilage xylan biosynthesis is diversified in different plant species. For example, IRX10 but not IRX9 or IRX14 might be responsible for the synthesis of the xylan backbone in psyllium mucilage because *IRX10* orthologues were highly presented in psyllium mucilage, while relatively very lower transcripts of *IRX9* and *IRX14* were detected in a transcriptome analysis [55].

## Conclusion

In this study, we functionally identified seven GT43 genes from *M. lutarioriparius.* Our results provided the first line of genetic evidence demonstrating that *Miscanthus* has evolved to retain two functionally non-redundant groups of GT43 genes involved in xylan biosynthesis. *MlGT43A-E* are functional orthologues of *IRX9*, while *MlGT43F* and *MlGT43G* are functional orthologues of *IRX14*. Nevertheless, functional divergence of *IRX14* orthologues in *M. lutarioriparius* has occurred as none of *MlGT43* genes could rescue the mucilage defects of *irx14* seeds. Furthermore, *MlGT43A-E* were differentially regulated by *SND1*, *MYB46* or *VND7* orthologues, the putative key regulators in secondary cell wall formation. The results obtained deepen our understanding of xylan biosynthesis in *Miscanthus*. Understanding how xylan polymers are synthesized may lay a foundation for the genetic modification of *Miscanthus* to be better suited for various economically important applications, including the more efficient utilization of xylan for biofuel production.

## Methods

### Plant materials and growth conditions

The *M. lutarioriparius* used in this study was provided by Shanghai Institute for Biological Sciences of the Chinese Academy of Sciences. The plants were clonally propagated by young rhizomes in greenhouse under 16 h light/8 h dark photoperiod at 25–28 °C.

T-DNA insertion mutants *irx9* (SALK_058238) and *irx14* (SALK_038212) were obtained from the *Arabidopsis* Biological Resource Center (ABRC). Seeds were surface sterilized and sowed on 1/2 MS plate. After stratified at 4 °C for 3 d, the plates were transferred to the growth chamber and germinated at 21 °C under 16 h light/8h dark photoperiod. Homozygous T-DNA insertions were identified by PCR of genomic DNA. The primers are listed in Additional file 7: Table S3.

### RNA isolation and Quantitative real-time RT-PCR (qRT-PCR) analysis

The total RNA was isolated from root, rhizome, stem, leaf and sheath of *M. lutarioriparius* using Trizol reagent (Invitrogen), then treated with RNase-free DNaseI (Promega) to remove genomic DNA contamination. First-strand cDNA was synthesized using M-MLV reverse transcriptase (TaKaRa, Japan) according to the manufacturer’s instructions. The cDNAs were used as templates for qRT-PCR with gene-specific primers (Additional file 7: Table S3). The qRT-PCR was carried out using LightCycler® 480 detection system (Roche) with SYBR® Premix Ex Taq II (TaKaRa). *MlACT11* was used as an internal control.

### Identification of *MlGT43* genes

The *Arabidopsis* GT43 proteins (IRX9, IRX9L, IRX14 and IRX14L) were used as baits to search against the draft genome sequence of *M. lutarioriparius* (Lu et al., unpublished data). Specific primers were designed to isolate the full length *MlGT43* cDNAs (Additional file 7: Table S3). The PCR products were purified, cloned into pMD19-T vector (TIANGEN) and sequenced. The exon/intron organization was illustrated with Gene Structure Display Server (GSDS) program (http://gsds.cbi.pku.edu.cn/) by alignment of the cDNAs with their corresponding genomic DNA sequences [56].

### Phylogenetic analysis of GT43 family from other plant species

GT43 family protein sequences from nine other species including moss (*P. patens*), spikemoss (*S. moellendorffii*), monocot angiosperms (*O. sativa*, *B. distachyon* and *S. bicolor*), and dicot angiosperms (*A. thaliana*, *P. trichocarpa*, *M. truncatula* and *V. vinifera*) were obtained using BLASTP search against Phytozome10 database (https://phytozome.jgi.doe.gov/). Phylogenetic analysis was performed with MEGA6.0 by the Neighbor-Joining (NJ) method with 1000 bootstrap replicates with default parameters [57].

### In situ mRNA hybridization

For the synthesis of antisense and sense probes, ~200 bp fragments of *MlGT43A-G* were amplified by PCR with their corresponding primers (Additional file 7: Table S3) and cloned into the pGM-T vector (TIANGEN). The RNA probes were synthesized with the DIG RNA labelling kit (Roche) according to the manufacturer’s instructions.

*Miscanthus* stem segments from the 11^th^ internode were fixed in FAA solution (70 % ethanol, 5 % formaldehyde and 5 % acetic acid) at 4 °C overnight, followed by dehydration in gradient ethanol series (10 % increments). The samples were embedded in paraplast and cut into 8 μm-thick sections. The sections were mounted onto slides, and hybridized with DIG-labeled antisense or sense RNA probes. Images were captured with the OLYMPUS BX51 microscope.

### Subcellular localization

The co-localization of fluorescent protein-tagged MlGT43A-G with the Golgi marker was examined using the tobacco leaf transient expression system [58]. The full-length *MlGT43* genes without a terminator codon were amplified and fused with yellow fluorescent protein (YFP) in pEarleyGate101 vector [59] via LR recombination reactions (Invitrogen). The proteins generated thus encode fusion proteins of MlGT43s with YFP tagged at the C terminus. After 3 days post co-infiltration of YFP fusion proteins and the Golgi marker into tobacco leaves, leaf epidermal cells were examined for yellow fluorescence signal using a FluoView FV1000 Laser Scanning confocal microscope (OLYMPUS) equipped with 488 nm argon laser.

### Overexpression vector construction and complementation

The full-length cDNA sequence of *MlGT43s* were amplified by PCR and ligated to the pGWC-T as described previously [60]. The products were sequenced and then transferred into the pEarleyGate 100 vector [59] via LR recombination reaction (Invitrogen) to produce the 35S CaMV overexpression constructs. The constructs were introduced into *Agrobacterium tumefaciens* strain EHA105 by electroporation.

For complementation analysis, the overexpression constructs were transformed into the *Arabidopsis irx9* heterozygous or *irx14* homozygous mutant via the floral dip method [61]. Positive T0 and T1 generation plants were screened by spraying BASTA solution (50 mg/L) onto one-week-old seedlings in soil. For *irx9* complemented lines, transformed seedlings were further genotyped with PCR to verify the homozygous T-DNA insertions. Homozygous T3 transgenic lines were used for further analysis.

### Microscopy and immunolocalization analysis

*Arabidopsis* inflorescence stems were taken 0.5 cm above the rosette of eight-week-old plants. Samples were fixed in FAA solution, dehydrated via a series of ethanol gradients, and embedded in paraplast. For light microscopy, 8 μm-thick sections were stained with 0.5 % (w/v) toluidine blue O (Sigma-Aldrich) for 2 min and rinsed with water. The sections were photographed with a BX51 light microscope (OLYMPUS).

For the immunolabelling, sections were incubated with the LM10 antibody (1/20 dilution) for 2 h, then washed three times with phosphate-buffered saline, followed by incubation with rabbit anti-rat Alexa Fluor488-conjugated secondary antibody (1/100 dilution) in the dark for 1 h. Images were captured using a BX51 light microscope (OLYMPUS) equipped with fluorescent light.

For transmission electron microscopy, samples were embedded in Spurr’s resin. Ultra-thin sections (70 nm) were viewed by a H-7650 electron microscope (HITACHI). Cell wall thickness was measured in metaxylem vessels and interfascicular fibres using the software SmileView (JEOL). For each construct, at least three transgenic lines with the most severe phenotypes were examined.

### Cell wall monosaccharide composition analysis

To prepare cell-wall alcohol-insoluble residues (AIR), eight-week-old inflorescence stems from at least 20 independent plants were collected, frozen in liquid nitrogen, and freeze-dried overnight using a lyophilizer. For monosaccharide composition analysis, AIR was hydrolyzed in 2 M trifluoroacetic acid for 2 h at 120 °C. The released monosaccharides were derived by 1-phenyl-3-methyl-5-pyrazolone (PMP) and the derivatives were separated on a Thermo ODS-2 C18 column (4.6 × 250 mm) connected to a Waters HPLC system. The absorbance was monitored at 245 nm. Cellulose content was assayed with the anthrone reagent according to Updegraff [62]. Lignin composition was determined using the acetyl bromide spectrophotometric method as described [63].

### Transcriptional activation analysis

The pBI221 vector was used to produce both effector and reporter constructs. The *MlSND1*, *MlMYB46a/b* and *MlVND7* effector constructs were obtained by PCR using *Miscanthus* stem cDNA as the template (Additional file 7: Table S3). All effector constructs were individually ligated between the CaMV 35S promoter and the NOS terminator after removing GUS from the pBI221 vector. The *MlGT43A-E* promoters were cloned by hiTAIL-PCR [64] and ligated upstream of the GUS reporter gene after removing the 35S promoter region of pBI221 to create the reporter constructs.

### Ethics approval and consent to participate

Not applicable.

### Consent for publication

Not applicable.

### Availability of data and materials

The data supporting the results of this article are included as additional files. The *MlGT43* gene and promoter sequences were deposited in the Genbank (https://www.ncbi.nlm.nih.gov/genbank) under accession numbers KX082754 to KX082765.

## Additional files

Additional file 1: Table S1. Sequence identity and similarity among seven MlGT43 proteins and their *Arabidopsis* orthologues. (DOCX 15 kb) Additional file 2: Protein sequences used for the phylogenetic analysis of GT43 family. (TXT 27 kb) Additional file 3: Figure S1. Cellulose and lignin contents in *MlGT43* complemented lines. Cell walls were prepared from pooled inflorescence stems of six independent plants per genotype and used for measurement of the contents of cellulose (A) and lignin (B). The data are means ± SE of three independent assays. (TIF 522 kb) Additional file 4: Figure S2. None of *MlGT43* genes could rescue the mucilage defect of *irx14* seeds. Seeds of WT (A), *irx14* (B) and *MlGT43A-G* complemented *irx14* lines (B-I) were stained by ruthenium red with gentle shaking for 30 min. Bar = 200 μm. (TIF 14007 kb) Additional file 5: Figure S3. Mucilage weight and monosaccharide composition of WT, *irx14* and *MlGT43* complemented *irx14* seeds. A, Mucilage weights from WT, *irx14* and *MlGT43* complemented *irx14* lines. Water-soluble and adherent mucilage were sequentially extracted with water and 2 M NaOH. Error bars indicate SD (*n* = 3). B and C, Monosaccharide composition of water-soluble and adherent mucilage from WT, *irx14* and *MlGT43* complemented *irx14* lines. (TIF 5994 kb) Additional file 6: Table S2. The cis-acting regulatory elements predicted in the promoter sequences of *MlGT43A-E*. (DOCX 20 kb) Additional file 7: Table S3. List of primers used in this study. (DOCX 18 kb)

## Abbreviations

IRX: irregular xylem
GT: glycosyltransferase
qRT-PCR: quantitative real-time RT-PCR
GX: (methyl)glucuronoxylan
AX: arabinoxylan
GAX: glucuronoarabinoxylan
GlcA: glucuronic acid
MeGlcA: methylglucuronic acid
Ara: arabinose
GUX: glucuronic acid substitution of xylan
GXMT: glucuronoxylan methyltransferase
TBL: trichome birefringence-like
XAT: xylan arabinosyltransferase
XAX: xylosyl arabinosyl substitution of xylan
Xyl: xylose
CDS: coding sequence
YFP: yellow fluorescent protein
WT: wild type
TBO: toluidine blue O
SND1: secondary wall-associated NAC domain protein 1
VND7: vascular-related NAC-domain 7
