# Supplementary material for: Functional conservation and divergence of Miscanthus lutarioriparius GT43 gene family in xylan biosynthesis
Source: BMC Plant Biol. 2016 Apr 26;16:102. doi: 10.1186/s12870-016-0793-5 (PMC4845329; doi:10.1186/s12870-016-0793-5)
Supplement: Additional file 1: Table S1. — Sequence identity and similarity among seven MlGT43 proteins and their Arabidopsis orthologues. (DOCX 15 kb) [file 12870_2016_793_MOESM1_ESM.docx]

**Additional file 1**

**Table S1.** Sequence identity and similarity among seven MlGT43 proteins and their *Arabidopsis* orthologues.

| **Symbol** | ***Arabidopsis* orthologue locus** | ***Arabidopsis* locus description** | **Score** | **E-value** | **Identity**  **(%)** | **Similarity**  **(%)** |
| --- | --- | --- | --- | --- | --- | --- |
| MlGT43A | AT2G37090.1 | AtIRX9 | 174 | 1e-43 | 37 | 56 |
| MlGT43B | AT2G37090.1 | AtIRX9 | 162 | 5e-40 | 41 | 56 |
| MlGT43C | AT1G27600.1 | AtIRX9L | 294 | 9e-80 | 42 | 59 |
| MlGT43D | AT1G27600.1 | AtIRX9L | 271 | 4e-73 | 48 | 70 |
| MlGT43E | AT1G27600.2 | AtIRX9L | 288 | 3e-78 | 53 | 71 |
| MlGT43F | AT5G67230.1 | AtIRX14L | 439 | e-124 | 59 | 78 |
| MlGT43G | AT5G67230.1 | AtIRX14L | 215 | 6e-56 | 37 | 52 |
