# Supplementary figures and images for: Functional conservation and divergence of Miscanthus lutarioriparius GT43 gene family in xylan biosynthesis
Source: BMC Plant Biol. 2016 Apr 26;16:102. doi: 10.1186/s12870-016-0793-5 (PMC4845329; doi:10.1186/s12870-016-0793-5)

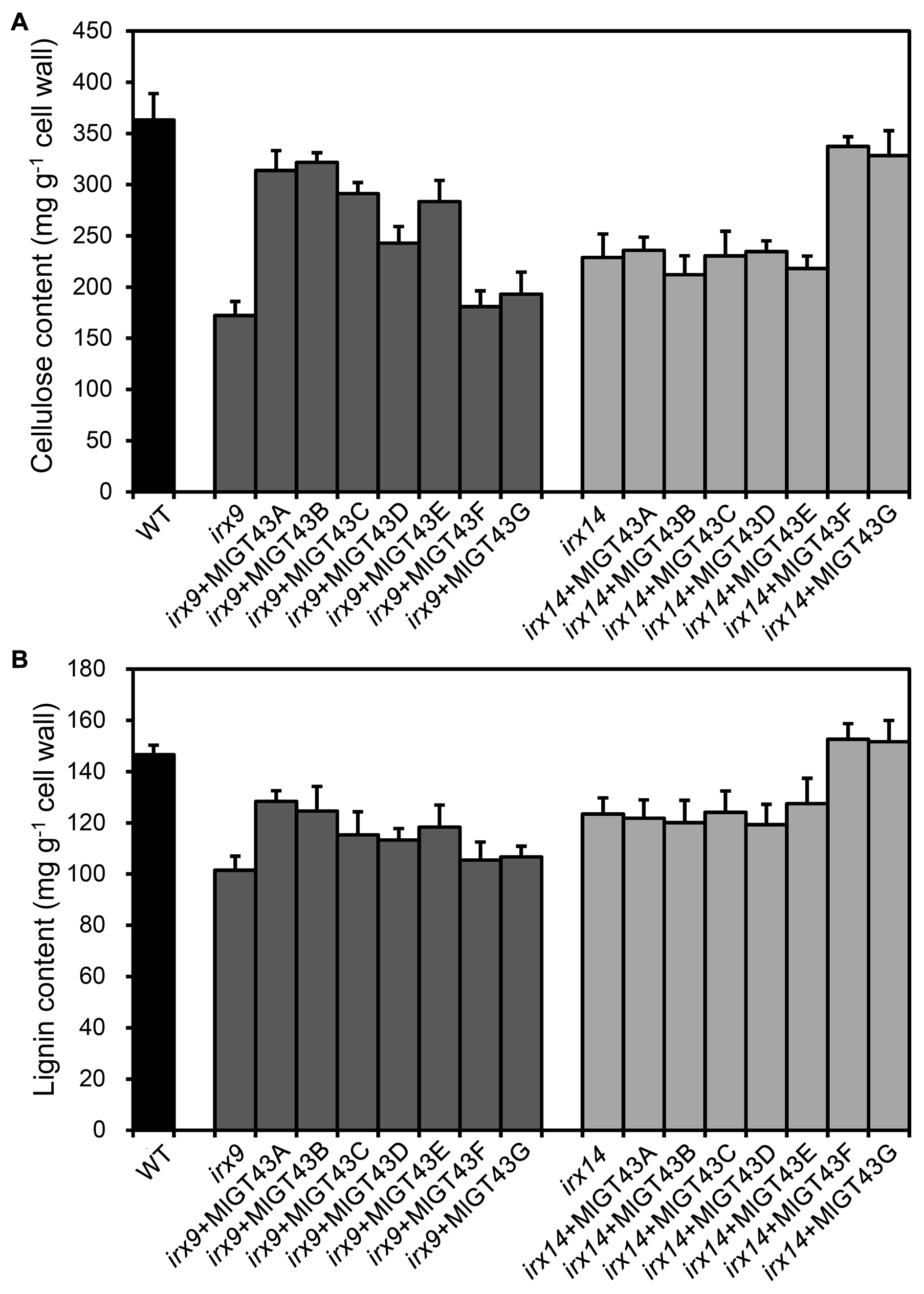

Supplement: Additional file 3: Figure S1. — Cellulose and lignin contents in MlGT43 complemented lines. Cell walls were prepared from pooled inflorescence stems of six independent plants per genotype and used for measurement of the contents of cellulose (A) and lignin (B). The data are means ± SE of three independent assays. (TIF 522 kb) [file 12870_2016_793_MOESM3_ESM.tif]

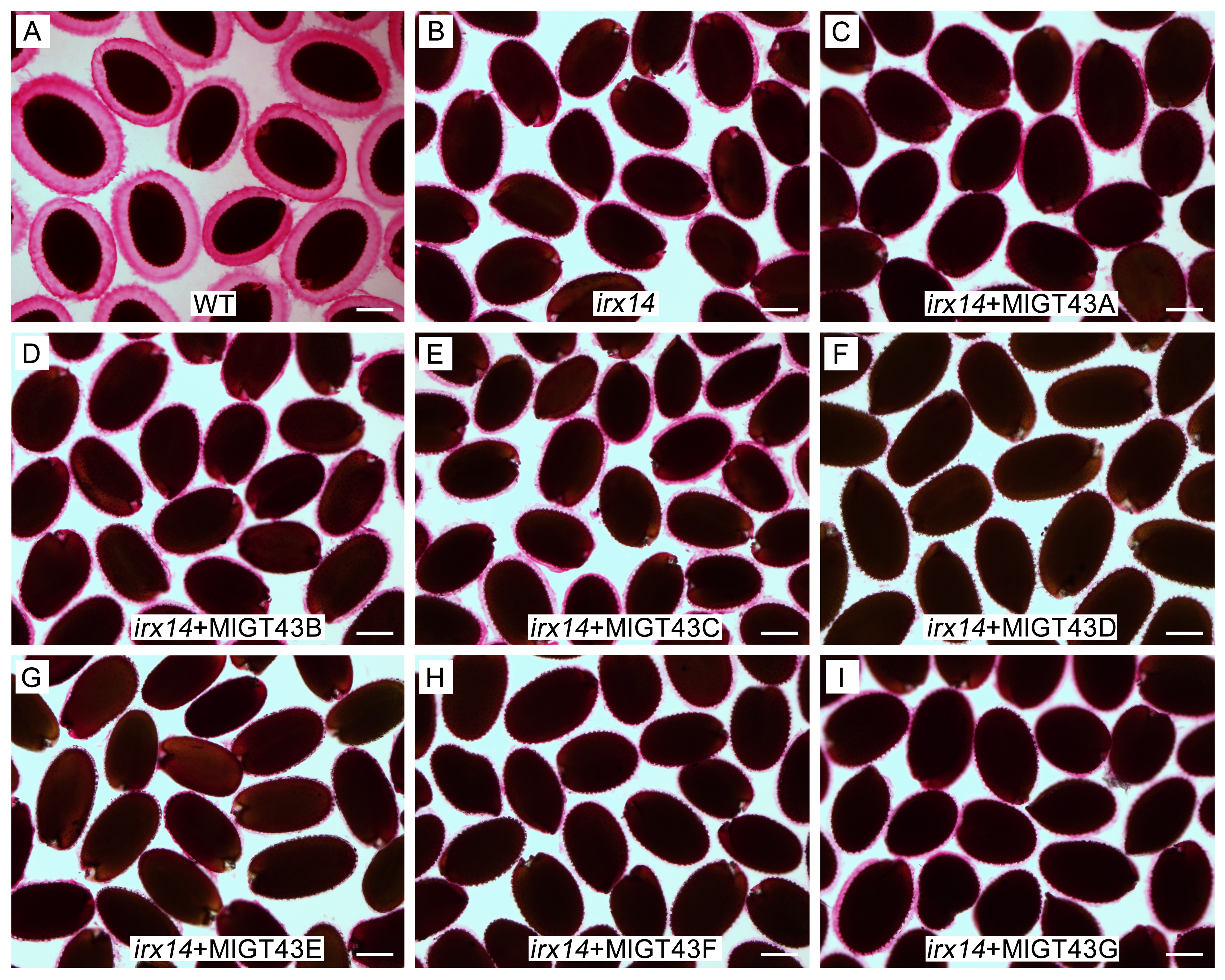

Supplement: Additional file 4: Figure S2. — None of MlGT43 genes could rescue the mucilage defect of irx14 seeds. Seeds of WT (A), irx14 (B) and MlGT43A-G complemented irx14 lines (B-I) were stained by ruthenium red with gentle shaking for 30 min. Bar = 200 μm. (TIF 14007 kb) [file 12870_2016_793_MOESM4_ESM.tif]

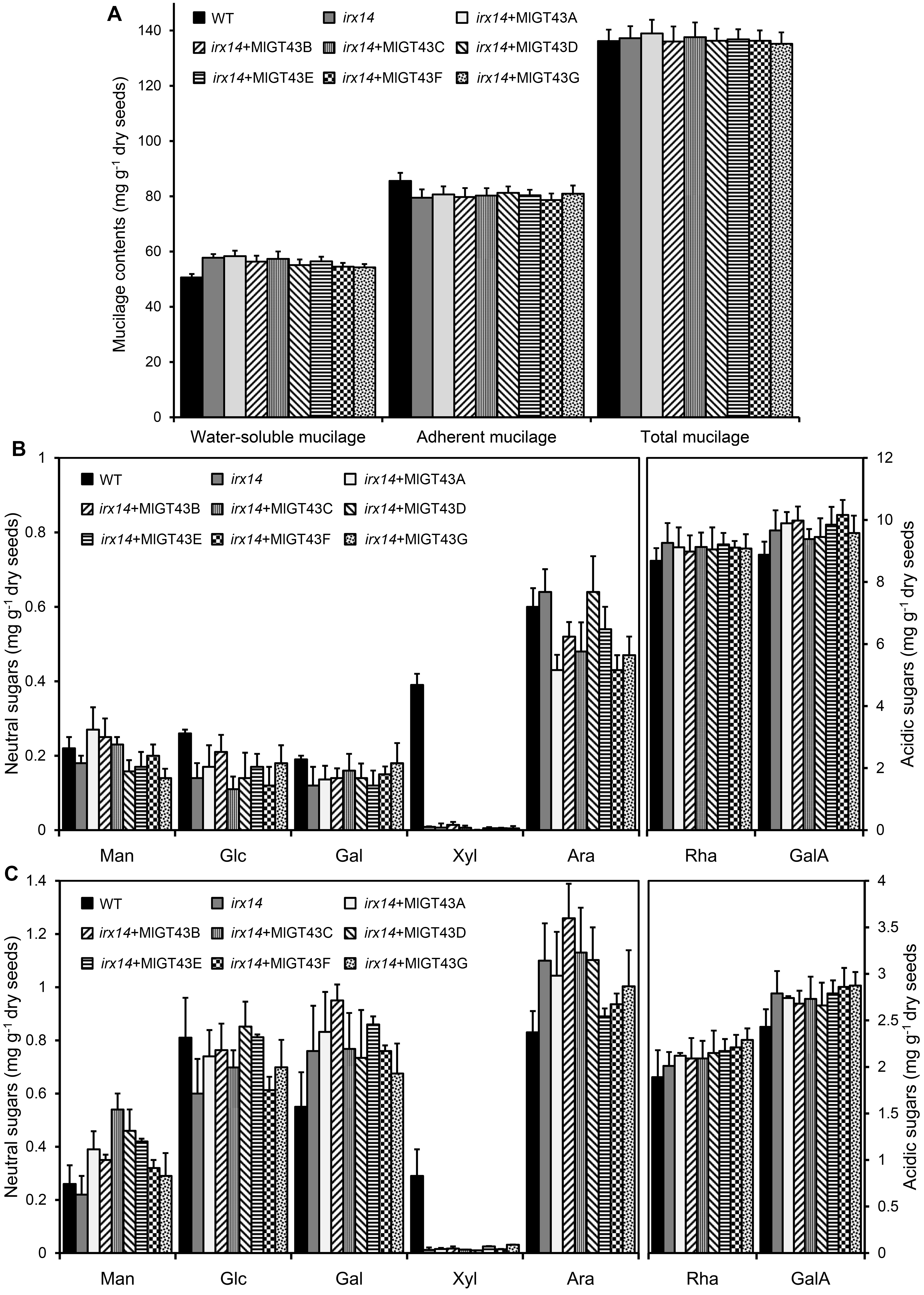

Supplement: Additional file 5: Figure S3. — Mucilage weight and monosaccharide composition of WT, irx14 and MlGT43 complemented irx14 seeds. A, Mucilage weights from WT, irx14 and MlGT43 complemented irx14 lines. Water-soluble and adherent mucilage were sequentially extracted with water and 2 M NaOH. Error bars indicate SD (n = 3). B and C, Monosaccharide composition of water-soluble and adherent mucilage from WT, irx14 and MlGT43 complemented irx14 lines. (TIF 5994 kb) [file 12870_2016_793_MOESM5_ESM.tif]
