# Supplementary material for: Functional conservation and divergence of Miscanthus lutarioriparius GT43 gene family in xylan biosynthesis
Source: BMC Plant Biol. 2016 Apr 26;16:102. doi: 10.1186/s12870-016-0793-5 (PMC4845329; doi:10.1186/s12870-016-0793-5)
Supplement: Additional file 6: Table S2. — The cis-acting regulatory elements predicted in the promoter sequences of MlGT43A-E. (DOCX 20 kb) [file 12870_2016_793_MOESM6_ESM.docx]

**Additional file 6**

**Table S2.** The cis-acting regulatory elements predicted in the promoter sequences of *MlGT43A-E*.

| ***MlGT43A***  **Promoter**  (1271 bp) | ***MlGT43B***  **Promoter**  (688 bp) | ***MlGT43C***  **Promoter**  (1055 bp) | ***MlGT43D***  **Promoter**  (827 bP) | ***MlGT43E***  **Promoter**  (1752 bp) | **Sequence** | **Description** |
| --- | --- | --- | --- | --- | --- | --- |
| TATA-box | TATA-box | TATA-box | TATA-box | TATA-box | ATATAAT | core promoter element around -30 of transcription start |
| CAAT-box | CAAT-box | CAAT-box | CAAT-box | CAAT-box | CCAAT | common cis-acting element in promoter and enhancer regions |
| 5’UTR Py-rich stretch | 5’UTR Py-rich stretch |  |  | 5’UTR Py-rich stretch | TTTCTTCTCT | cis-acting element conferring high transcription levels |
| ARE | ARE |  | ARE | ARE | TGGTTT | cis-acting regulatory element essential for the anaerobic induction |
|  | ABRE | ABRE |  | ABRE | GCAACGTGTC | cis-acting element involved in the abscisic acid responsiveness |
| AT1-motif | AT1-motif |  |  |  | AATTATTTTTTATT | part of a light responsive module |
|  |  | A-box | A-box | A-box | CCGTCC | cis-acting regulatory element |
|  |  | CCGTCC-box | CCGTCC-box |  | CCGTCC | cis-acting regulatory element related to meristem specific activation |
| HSE | HSE | HSE |  | HSE | AAAAAATTTC | cis-acting element involved in heat stress responsiveness |
| MBS | MBS |  | MBS | MBS | CAACTG | MYB binding site involved in drought-inducibility |
| MRE | MRE |  |  |  | AACCTAA | MYB binding site involved in light responsiveness |
| ATCT motif | ATCT-motif |  |  |  | AATCTAATCC | part of a conserved DNA module involved in light responsiveness |
|  | Sp1 | Sp1 | Sp1 | Sp1 | CC(G/A)CCC | light responsive element |
| G-box | G-box | G-box | G-box |  | CACGTT | cis-acting regulatory element involved in light responsiveness |
| Skn-1_motif | Skn-1_motif |  |  | Skn-1_motif | GTCAT | cis-acting regulatory element required for endosperm expression |
| GAG-motif |  | GAG-motif |  | GAG-motif | GAGAGAT | part of a light responsive element |
|  | Box 4 |  |  | Box 4 | ATTAAT | part of a conserved DNA module involved in light responsiveness |
|  | TGACG-motif | TGACG-motif | TGACG-motif |  | TGACG | cis-acting regulatory element involved in the MeJA-responsiveness |
|  | LTR |  | LTR | LTR | CCGAAA | cis-acting element involved in low-temperature responsiveness |
|  | CGTCA-motif | CGTCA-motif | CGTCA-motif |  | CGTCA | cis-acting regulatory element involved in the MeJA-responsiveness |
| I-box |  | I-box |  | I-box | GATATGG | part of a light responsive element |
| TC-rich repeats |  | TC-rich repeats |  |  | ATTTTCTTCA | cis-acting element involved in defense and stress responsiveness |
| P-box |  |  | P-box |  | CCTTTTG | gibberellin-responsive element |
| TGA-element |  |  |  | TGA-element | AACGAC | auxin-responsive element |
| TCA-element |  |  | TCA-element |  | CAGAAAAGGA | cis-acting element involved in salicylic acid responsiveness |
|  |  | chs-Unit 1 m1 |  | chs-Unit 1 m1 | ACCTAACCCGG | part of a light responsive element |
|  | GCN4_motif |  | GCN4-motif | GCN4-motif | TGAGTCA | cis-regulatory element involved in endosperm expression |
|  |  | GC-motif |  | GC-motif | CCCCCG | enhancer-like element involved in anoxic specific inducibility |
|  |  | CAT-box |  | CAT-box | GCCACT | cis-acting regulatory element related to meristem expression |
|  |  | TCCC-motif | TCCC-motif |  | TCTCCCT | part of a light responsive element |
| Box-W1 |  | Box-W1 |  | Box-W1 | TTGACC | fungal elicitor responsive element |
| GT1-motif | GT1-motif |  |  |  | GGTTAA | light responsive element |
| W box |  | W box |  | W box | TTGACC |  |
|  |  | AC-I |  | AC-I | CCCACCTACC |  |
|  |  | AC-II |  | AC-II | CCACCAACCCCC |  |
| unnamed-1 | unnamed-1 | unnamed-1 | unnamed-1 | unnamed-1 | CGTGG |  |
| unnamed-3 | unnamed-3 | unnamed-3 | unnamed-3 | unnamed-3 | CGTGG |  |
| unnamed-4 | unnamed-4 | unnamed-4 | unnamed-4 | unnamed-4 | CTCC |  |
| circadian |  |  |  |  | CAANNNNATC | cis-acting regulatory element involved in circadian control |
| EIRE |  |  |  |  | TTCGACC | elicitor-responsive element |
| unnamed-11 |  |  |  |  | TCCACATAGA |  |
|  | TCT-motif |  |  |  | TCTTAC | part of a light responsive element |
|  | GARE-motif |  |  |  | AAACAGA | gibberellin-responsive element |
|  | ACE |  |  |  | GACACGTATG | cis-acting element involved in light responsiveness |
|  | o2-site |  |  |  | GATGACATGG | cis-acting regulatory element involved in zein metabolism regulation |
|  |  | CATT-motif |  |  | GCATTC | part of a light responsive element |
|  |  | AT-rich element |  |  | ATAGAAATCAA | binding site of AT-rich DNA binding protein (ATBP-1) |
|  |  | 3-AF1 binding site |  |  | AAGAGATATTT | light responsive element |
|  |  | rbcS_CMA7a |  |  | GTCGATAAGG | part of a light responsive element |
|  |  | AuxRR-core |  |  | GGTCCAT | cis-acting regulatory element involved in auxin responsiveness |
|  |  | CCAAT-box |  |  | CAACGG | MYBHv1 binding site |
|  |  | AAGAA-motif |  |  | GAAAGAA |  |
|  |  | TCCACCT-motif |  |  | TCCACCT |  |
|  |  | TATCCAT/C-motif |  |  | TATCCAT |  |
|  |  | plant_AP-2-like |  |  | CGCGCCGG |  |
|  |  | unnamed_8 |  |  | CATTTTTGT |  |
|  |  |  | Box II |  | ACACGTTGT | part of a light responsive element |
|  |  |  | WUN-motif |  | TCATTACGAA | wound-responsive element |
|  |  |  | GA-motif |  | AAGGAAGA | part of a light responsive element |
|  |  |  | box S |  | AGCCACC |  |
|  |  |  | unnamed-2 |  | CCCCGG |  |
|  |  |  |  | RY-element | CATGCATG | cis-acting regulatory element involved in seed-specific regulation |
|  |  |  |  | GATA-motif | GATAGGA | part of a light responsive element |
|  |  |  |  | GATT-motif | CTCCTGATTGGA | part of a light responsive element |
|  |  |  |  | GCC box | AGCCGCC |  |
