# Supplementary material for: Functional conservation and divergence of Miscanthus lutarioriparius GT43 gene family in xylan biosynthesis
Source: BMC Plant Biol. 2016 Apr 26;16:102. doi: 10.1186/s12870-016-0793-5 (PMC4845329; doi:10.1186/s12870-016-0793-5)
Supplement: Additional file 7: Table S3. — List of primers used in this study. (DOCX 18 kb) [file 12870_2016_793_MOESM7_ESM.docx]

**Additional file 7**

**Table S3.** List of primers used in this study.

|  | **Primer name** | **Primer Sequence (5'-3')** |
| --- | --- | --- |
| Genotyping primers for T-DNA insertion mutants | SALK_058238_LP | CCAAAACTGTCAATTTATAACATTGG |
|  | SALK_058238_RP | ATGTTCAATGTGCCTCAAAGC |
|  | SALK_038212_LP | AACGACACGTGTACCTCCTTG |
|  | SALK_038212_RP | AACATCACAATCCCATCAAGC |
| Gene cloning | *MlGT43A*_1F | ATGGAGCCAGCAGGGAGG |
|  | *MlGT43A*_1083R | TCATGTTGTGGGATCTTCATCG |
|  | *MlGT43B*_1F | ATGGCCTCGCCCAAGCATTCT |
|  | *MlGT43B*_1134R | CTAGAGCAGCGTCGTGTCGA |
|  | *MlGT43C*_1F | ATGGCCCGAAGAAATGCC |
|  | *MlGT43C*_1347R | TTATGTTATAGGCACGATGATGTCTA |
|  | *MlGT43D*_1F | ATGGCGTCCAGTCGAAGGA |
|  | *MlGT43D*_1356R | CTATATTACAGGCATGTCAGCTTCC |
|  | *MlGT43E*_1F | ATGGCGTCGATCCGCCG |
|  | *MlGT43E*_1161R | CTATTTAAGAGGAATGATCA |
|  | *MlGT43F*_1F | ATGCGCGCGCACCGGATCAT |
|  | *MlGT43F*_1077R | TCAGTTTTCTTGCCCTTGAGG |
|  | *MlGT43G*_1F | ATGGCCGTCGGCTTCCG |
|  | *MlGT43G*_1227R | CTAGTGCTGGTCTTCAGTGCTT |
|  | *MlMYB46a*_1F | ATGGGGAGGCACTCCTGCTGTTA |
|  | *MlMYB46a*_1284R | CTAGATATTTTCAAAAGACAAGGAC |
|  | *MlMYB46b*_1F | ATGGGGAGGCATTCTTGCTGC |
|  | *MlMYB46b*_1293R | CTAGATATTCTCAAAAGACAGTTGC |
|  | *MlVND7*_1F | ATGGAATCATGCGTGCCCCC |
|  | *MlVND7*_1104R | TCATTTCTCGAACACACACAGTCC |
|  | *MlSND1*_1F | ATGAGCATCTCGGTGAACGGG |
|  | *MlSND1*_1263R | CTAGACGTTGTTCATCGTCAAGTCC |
| Expression analysis | *MlGT43A*_716F | CAGATACCAATACCACCGCAAAC |
|  | *MlGT43A*_793R | TCCTGAACCCAATTCCAGAGATG |
|  | *MlGT43B*_860F | TCTCAGTCTCAGCCTCGGAAG |
|  | *MlGT43B*_1056R | GTAGTCTGCCACCACCAACC |
|  | *MlGT43C*_165F | GATGTCCAGCAGCCTCAAGTC |
|  | *MlGT43C*_295R | CCAAGAAGCACACAAGCAGATG |
|  | *MlGT43D*_208F | CAGAGGCATAGTAGATCACAGGAG |
|  | *MlGT43D*_333R | GACAGAGAACAAAGGCATGAACC |
|  | *MlGT43E*_783F | GGATGGTGTGGTGCTGGAAG |
|  | *MlGT43E*_911R | ATGGTGCTGTTGAACGCGAAAC |
|  | *MlGT43F*_829F | CTGTGGTGGCTCCGTGTTG |
|  | *MlGT43F*_995R | CTATCCTCCTGCTCTTGCTTGTC |
|  | *MlGT43G*_1075F | TCTCTGCGTTCCGACAGTCTC |
|  | *MlGT43G*_1225R | AGTGCTGGTCTTCAGTGCTTGCT |
|  | *MlACT11*_644F | CCGTGAGAAGATGACCCAGAT |
|  | *MlACT11*_971F | GTCGTAGTCCAGGGCAATGTAG |
|  | *AtUBQ10_F* | GGCCTTGTATAATCCCTGATGAATAAG |
|  | *AtUBQ10_R* | AAAGAGATAACAGGAACGGAAACATAGT |
| In situ hybridization | *MlGT43A*_719F | ATACCAATACCACCGCAAACA |
|  | *MlGT43A*_962R | TCCATGTGCCACAACATTACC |
|  | *MlGT43B*_155F | TGTGCTTCCTGGTGGGTCTCC |
|  | *MlGT43B*_348R | CTGCTGGTACGACTGGTAGTGCTG |
|  | *MlGT43C*_508F | TTGGATGATGAGGCGGACTT |
|  | *MlGT43C*_722R | GTTTCACGGGACTGATACGG |
|  | *MlGT43D*_201F | GACTTCCCAGAGGCATAG |
|  | *MlGT43D*_408R | CATCGTTTCCGTCTCAAT |
|  | *MlGT43E*_257F | GCGTCTACTCCCTCGACCTCT |
|  | *MlGT43E*_457R | CGAAACCCGACATAGCAACAT |
|  | *MlGT43F*_241F | TCGTCCACATCCCATTCC |
|  | *MlGT43F*_452R | GATTCCAACGGATACAGCAC |
|  | *MlGT43G*_921F | GCTGTGGGAGGACACCAAGGA |
|  | *MlGT43G*_1153R | CACCGTTATTCAGCAGTGGAGGA |
| Promoter cloning | *MlGT43A*_F | CCAAAACCTAAGAGAAAGAGAG |
|  | *MlGT43A*_R | CACCAGCAGCTAGCAAGCTAGT |
|  | *MlGT43B*_F | TGGCGGTTGAACCCCACTGT |
|  | *MlGT43B*_R | GGCGCTACCTAGCTCGG |
|  | *MlGT43C*_F | TGGTCGGTAGGGCTTGGCT |
|  | *MlGT43C*_R | AGCAGGAGCACAGCACCG |
|  | *MlGT43D*_F | GCACAATGGGAACAGCTCA |
|  | *MlGT43D*_R | TCCAGAATCAAGGCTTCGTG |
|  | *MlGT43E*_F | GTCGTCAACCCGCTGCTGT |
|  | *MlGT43E*_R | TATTGTTGTTCGTTGCCGG |
